# Artificial Intelligence-Enabled Analysis of WNT Pathway Dysregulation in Bevacizumab-Treated Early-Onset Colorectal Cancer

**DOI:** 10.3390/ijms27146195

**Published:** 2026-07-11

**Authors:** Erika Ruiz-Garcia, Brigette Waldrup, Francisco G. Carranza, Sophia Manjarrez, Edith A. Fernandez-Figueroa, Enrique Velazquez-Villarreal

**Affiliations:** 1 Translational Medicine Laboratory, GI Oncology Department, INCAN—Instituto Nacional de Cancerología, Ciudad de México 14080, Mexico; 2 Department of Integrative Translational Sciences, Beckman Research Institute, City of Hope, Duarte, CA 91010, USA; 3 Núcleo B de Innovación en Medicina de Precisión, Instituto Nacional de Medicina Genómica, Ciudad de México 14610, Mexico; 4 Comprehensive Cancer Center, City of Hope, Duarte, CA 91010, USA

**Keywords:** colorectal cancer, precision oncology, bevacizumab, artificial intelligence, LLM, conversational AI, AI-Agents, WNT pathway

## Abstract

Early-onset colorectal cancer (EOCRC) is increasing disproportionately among Hispanic/Latino (H/L) populations and demonstrates substantial molecular and clinical heterogeneity. Although Wingless/Integrated (WNT) pathway alterations are among the most common genomic events in colorectal cancer, their prognostic significance in the context of contemporary systemic therapies, including Bevacizumab, remains incompletely understood. We conducted an integrative clinical–genomic analysis of colorectal cancer cohorts stratified by age at diagnosis, ancestry, and Bevacizumab exposure, interrogating somatic alterations across curated WNT signaling pathway genes. Conversational artificial intelligence agents (AI-HOPE and AI-HOPE-WNT) enabled dynamic cohort construction, treatment-specific subgroup analyses, and pathway-level interrogation through natural language-driven clinical–genomic integration. WNT pathway alterations, predominantly involving APC, were highly prevalent across all cohorts; however, their distribution and clinical associations demonstrated strong treatment-, ancestry-, and age-dependent variability. Bevacizumab-treated tumors exhibited lower mutation frequencies in several WNT regulators, including RNF43, AXIN1/2, TCF7L2, and AMER1, suggesting potential biologic interaction or treatment-related selective pressure. Importantly, WNT pathway alterations were associated with improved overall survival in H/L EOCRC and Non-Hispanic White (NHW) late-onset colorectal cancer, but with worse survival in NHW EOCRC, highlighting distinct ancestry- and age-specific prognostic effects. These findings support the role of the WNT pathway dysregulation as a disparity-aware biomarker framework in colorectal cancer and demonstrate the utility of conversational AI systems for scalable multidimensional clinical–genomic integration in precision oncology.

## 1. Introduction

Colorectal cancer (CRC) is the third most commonly diagnosed malignancy and the second leading cause of cancer-related mortality worldwide [1]. Despite advances in screening and treatment, early-onset CRC (EOCRC; diagnosis < 50 years) is rising by approximately 1–2% annually [2,3]. EOCRC has transitioned from the fourth to the leading cause of cancer-related death in men and the second in women under 50 [2,3]. Because routine screening typically begins at age 50, EOCRC is often diagnosed at advanced stages, contributing to poorer outcomes.

These trends are exacerbated by racial and ethnic disparities. Although Non-Hispanic Whites (NHWs) have the highest absolute CRC incidence in the United States, Hispanic/Latino (H/L) populations experience the fastest increase in EOCRC, with annual incidence rising by 2.35% [4,5,6,7]. Yet, genomic studies disproportionately focus on NHWs, limiting understanding of EOCRC biology in H/L patients—an important gap, given evidence of reduced therapeutic benefit and poorer outcomes in this population [8].

EOCRC may represent a biologically distinct disease entity. Prior studies report higher microsatellite instability, increased tumor mutation burden, elevated PD-L1 expression, greater prevalence of hereditary CRC syndromes, and reduced LINE-1 methylation compared with later-onset CRC [9,10,11,12,13,14]. Genomic analyses across key CRC pathways, including TP53, KRAS, WNT, and TGF-β, further reveal ancestry-linked differences in tumor biology, particularly among underserved populations such as H/L patients [15,16,17,18,19].

The biological complexity of CRC at the intersection of age, ancestry, and treatment exposure challenges traditional analytic approaches. Large clinical–genomic datasets are heterogeneous and difficult to interrogate using static models, underscoring the need for advanced, interpretable computational frameworks capable of pathway-level inference across clinically meaningful subgroups.

Among CRC signaling pathways, WNT plays a central role in tumor initiation and progression. Canonical WNT signaling promotes β-catenin stabilization and transcriptional activation of oncogenic programs [20,21,22], with APC mutations occurring in approximately 80% of CRCs [23]. However, microsatellite-stable EOCRC appears to harbor fewer WNT alterations than later-onset disease, suggesting potential differences in pathway activation and therapeutic vulnerability [13].

Clinically, EOCRC is more frequently diagnosed at advanced stages and is associated with poorer outcomes [24,25]. Bevacizumab, an anti-VEGF-A monoclonal antibody, is a cornerstone of first-line therapy for metastatic CRC and improves survival when combined with standard chemotherapy [26,27,28,29,30,31,32]. Notably, therapeutic benefit and toxicity profiles may vary by age and ancestry, with underrepresented populations—including H/L patients—exhibiting differential responses [33,34].

Emerging evidence suggests biologic crosstalk between WNT and VEGF signaling. Preclinical studies indicate that WNT pathway activation influences angiogenesis, endothelial remodeling, and resistance to anti-angiogenic therapy [29,35,36]. Despite these insights, no clinical study has systematically evaluated the impact of WNT pathway alterations on bevacizumab outcomes in EOCRC, particularly in H/L patients who remain underrepresented in genomic research.

Given the rising burden of EOCRC in H/L populations, the central role of WNT dysregulation in CRC, and the widespread use of bevacizumab, a focused investigation of WNT pathway alterations in bevacizumab-treated EOCRC across ancestries is warranted [37]. Here, we integrate multi-cohort clinical, genomic, and treatment data to characterize WNT alterations, assess their association with bevacizumab exposure, and identify ancestry-specific molecular signatures using conversational AI-enabled [38,39] precision oncology [40] frameworks (Figure S1).

## 2. Results

### 2.1. Comparison of Clinical and Demographic Characteristics in Hispanic/Latino vs. Non-Hispanic White CRC Cohorts

The cohort included 275 H/L and 2442 NHW patients with primary colorectal adenocarcinoma, as shown in Table 1. H/L patients showed a higher proportional burden of early-onset CRC, while bevacizumab use was more frequent in late-onset disease across both ancestries. Tumor classification differed by ancestry, with most H/L cases annotated as colorectal adenocarcinoma NOS (96.7%), compared with greater colon and rectal subtype diversity in NHW patients. Sex distribution, tumor availability, and stage at diagnosis were similar between groups (~60% stage I–III; ~40% stage IV). Bevacizumab exposure was modestly higher, and treatment data was more frequently missing among H/L patients, underscoring the importance of ancestry-aware analyses when interpreting molecular and therapeutic associations in CRC.

### 2.2. Comparative Genomic Analysis by Age and Ancestry

Among H/L patients, age at diagnosis did not differ by bevacizumab exposure in either EOCRC or LOCRC. However, bevacizumab-treated EOCRC tumors exhibited significantly lower tumor mutational burden (TMB), alongside a higher fraction of genome-altered (FGA) and chromosomal instability, compared with untreated EOCRC. RNF43 mutations were absent in treated EOCRC and markedly reduced in treated LOCRC, but more frequent in untreated tumors across age groups, as shown in Table 2.

Across both EOCRC and LOCRC, bevacizumab exposure was generally associated with lower mutation burden, reduced TMB, and decreased frequencies of several key WNT pathway regulators, including RNF43, AXIN1/2, and TCF7L2 (Table 2 and Table S1).

Notably, among Hispanic/Latino patients with LOCRC, RNF43 alterations were significantly less frequent in bevacizumab-treated tumors compared with untreated tumors (*p* = 0.0086), suggesting a potential interaction between bevacizumab exposure and the molecular landscape of WNT signaling in this population (Table 2).

Ancestry-based comparisons in EOCRC revealed no differences in age, mutation burden, or TMB between bevacizumab-treated H/L and NHW patients; however, H/L tumors exhibited significantly higher FGA. In untreated EOCRC, H/L patients were diagnosed earlier than NHW patients and showed a trend toward higher mutation burden, while TMB and FGA were comparable, as shown in Table S1.

### 2.3. Distribution of WNT Pathway Alterations by Age, Ethnic Background, and Exposure to Bevacizumab

WNT pathway alterations were highly prevalent across all colorectal cancer subgroups, with minimal variation by age of onset, ancestry, or Bevacizumab exposure (Table S2).

In H/L patients, WNT alterations were common in both EOCRC and LOCRC, with similar frequencies between Bevacizumab-treated and untreated groups (EOCRC: 86.7% vs. 91.2%, *p* = 0.49; LOCRC: 83.7% vs. 84.7%, *p* = 1.00). Likewise, in NHW patients, WNT alterations were frequent across EOCRC and LOCRC, with no meaningful differences by treatment status (EOCRC: 82.0% vs. 87.7%, *p* = 0.0736; LOCRC: 88.9% vs. 88.5%, *p* = 0.88).

Comparisons by ancestry showed similarly high WNT alteration rates in both EOCRC and LOCRC, regardless of Bevacizumab exposure, with low proportions of alteration-negative tumors across all strata (≈9–18%). Overall, these findings indicate that WNT pathway dysregulation is a ubiquitous feature of colorectal cancer, largely independent of age, ancestry, or Bevacizumab treatment.

### 2.4. Frequencies of Gene Alterations in the WNT Pathway

Across ancestry, age, and treatment strata, APC was the dominant WNT alteration (≈ 65–83%) and did not differ significantly by Bevacizumab exposure, reinforcing its role as a shared canonical driver (Tables S3–S14). In contrast, non-canonical WNT regulators, particularly RNF43, AXIN1/2, TCF7L2, and AMER1, showed treatment- and age-dependent variation.

In H/L EOCRC and LOCRC, RNF43 mutations were consistently enriched in untreated tumors and depleted in Bevacizumab-treated cases, reaching significance in both EOCRC (14.3% vs. 0%; Table S3) and LOCRC (18.0% vs. 2.3%; Table S4). Among Bevacizumab-treated H/L patients, EOCRC and LOCRC exhibited nearly identical WNT mutation profiles, with rare secondary alterations and no age-specific differences (Table S5), whereas untreated EOCRC showed significantly higher APC mutation frequency than LOCRC (Tables S6 and S7).

In NHW patients, Bevacizumab exposure was associated with broad depletion of non-canonical WNT alterations, most prominently in LOCRC, where AXIN1, AXIN2, RNF43, TCF7L2, and AMER1 mutations were significantly reduced in treated tumors (Tables S8 and S9). In untreated NHW patients, EOCRC was enriched for APC mutations, while LOCRC showed higher frequencies of non-canonical regulators, indicating an age-related shift in WNT architecture (Table S10).

Cross-ancestry comparisons revealed highly similar WNT mutation frequencies in Bevacizumab-treated EOCRC and LOCRC, with no significant differences between H/L and NHW patients (Tables S11–S13). In untreated disease, ancestry-associated differences were modest and largely non-significant, with the exception of slightly higher APC mutation frequency in untreated NHW LOCRC (Table S14).

### 2.5. Mutational Landscape

**Figure 1:**
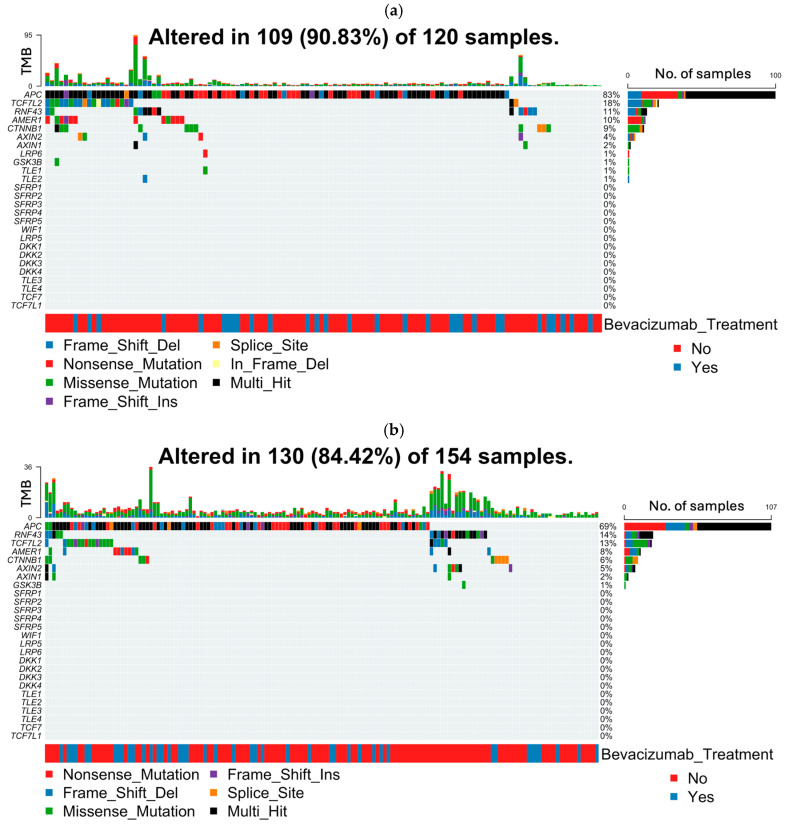

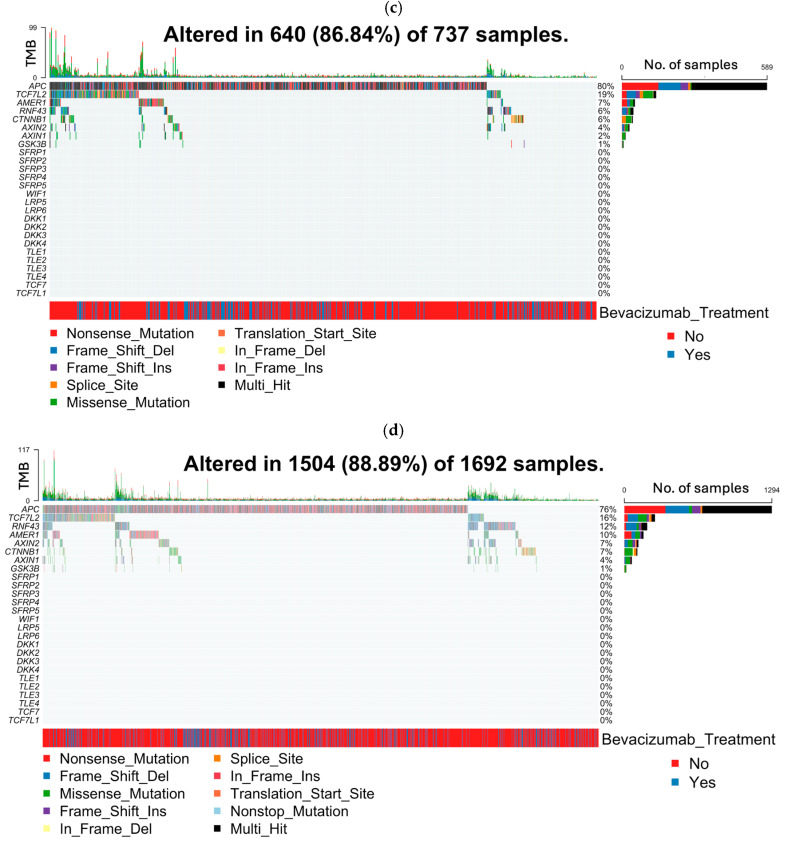
Somatic mutation profiles of WNT pathway genes in colorectal cancer (CRC) stratified by age and ancestry. Oncoplots illustrate gene-level alteration patterns across WNT pathway components in CRC, separated by early- vs. late-onset disease and by H/L vs. NHW ancestry. Each panel displays mutation classes, tumor mutational burden (TMB), and Bevacizumab treatment status for: (**a**) 120 early-onset H/L patients, (**b**) 154 late-onset H/L patients, (**c**) 737 early-onset NHW patients, and (**d**) 1692 late-onset NHW patients. APC emerges as the most frequently altered gene across all groups, dominated by truncating variants. Additional recurrent mutations in RNF43, TCF7L2, AMER1, and CTNNB1 underscore widespread disruption of WNT signaling, with age- and ancestry-related variation evident in the mutational patterns.

Across all ancestry and age groups, WNT pathway alterations were highly prevalent (≥84%), underscoring WNT dysregulation as a core feature of CRC (Figure 1; Table S15).

In EOCRC H/L patients, nearly all tumors harbored at least one WNT alteration (Figure 1a). APC was the dominant mutated gene, enriched for truncating events, followed by RNF43, AMER1, CTNNB1, and TCF7L2, each showing heterogeneous variant types. Tumor mutational burden (TMB) was generally low, with a small subset of hypermutated cases. Bevacizumab-treated and untreated tumors were intermixed without distinct mutational clustering (Figure 1a; Table S15).

Late-onset H/L CRC similarly showed frequent WNT alterations (84.4%), with APC and RNF43 most commonly affected, alongside recurrent AMER1 and TCF7L2 mutations (Figure 1b; Table S13). TMB remained largely low, and mutation patterns did not segregate by bevacizumab exposure.

Among NHW patients, WNT alterations were observed in 86.8% of EOCRC and 88.9% of LOCRC tumors (Figure 1c,d). APC truncations predominated across both age groups, with recurrent alterations in TCF7L2, RNF43, AMER1, and CTNNB1. A minority of cases exhibited elevated TMB, consistent with hypermutator phenotypes. Bevacizumab-treated and untreated tumors showed substantial overlap in mutation profiles across age strata (Figure 1c,d; Table S15).

### 2.6. Survival Analysis

Kaplan–Meier analyses assessed associations between WNT pathway alterations and overall survival across colorectal cancer subgroups stratified by age, ancestry, and Bevacizumab treatment.

**Figure 2:**
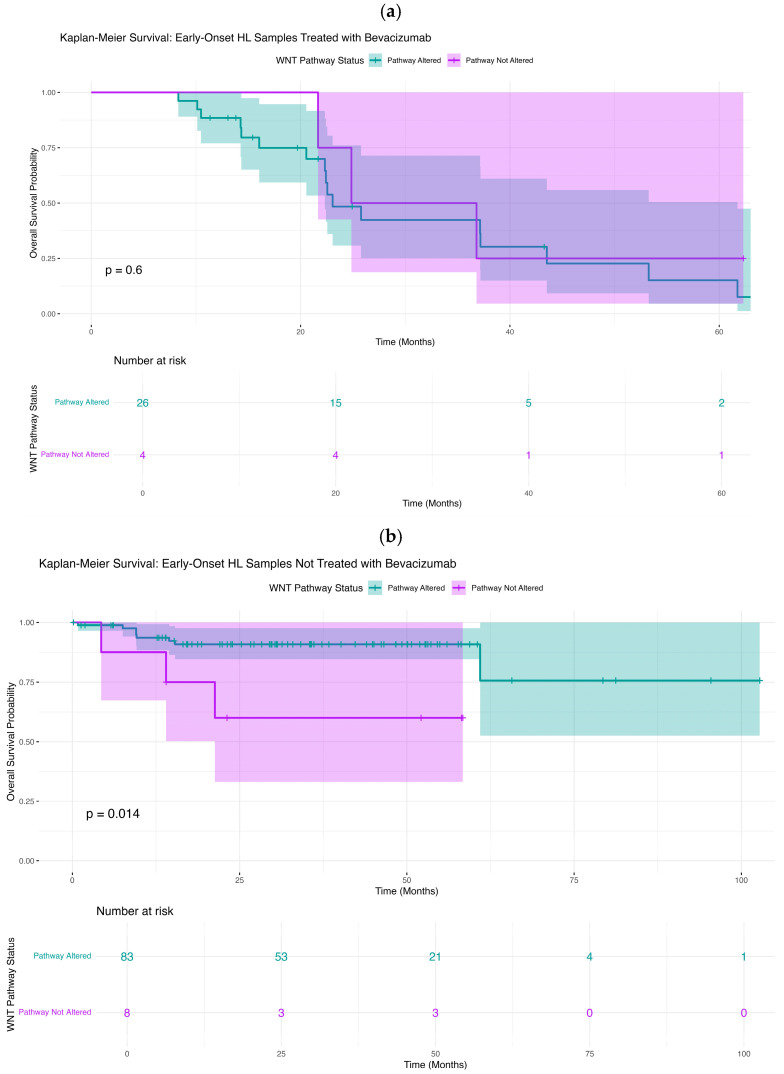

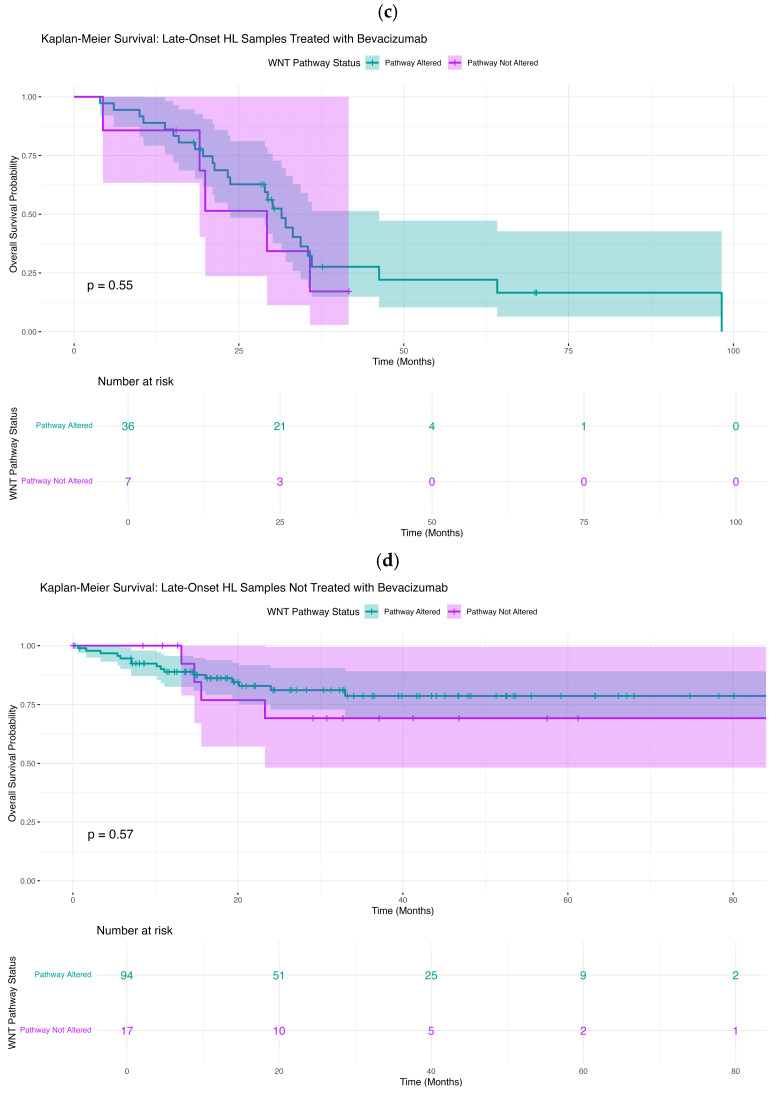

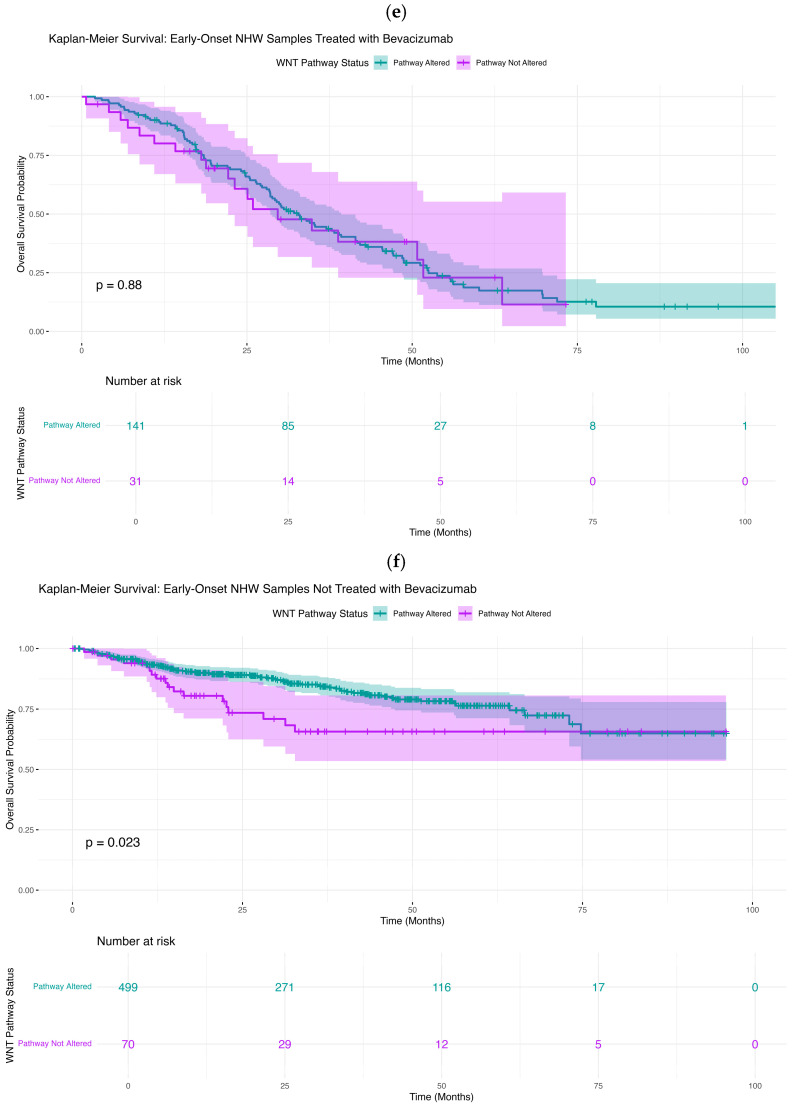
Kaplan–Meier survival analysis according to WNT pathway alteration status across age and Bevacizumab treatment status in colorectal cancer. Overall survival analyses were performed across patient groups: (**a**) late-onset NHW patients treated with Bevacizumab, and (**b**) late-onset NHW patients not receiving Bevacizumab. (**a**) Early-onset Hispanic/Latino (H/L) treated with Bevacizumab; (**b**) early-onset H/L without Bevacizumab; (**c**) late-onset H/L treated with Bevacizumab; (**d**) late-onset H/L without Bevacizumab; (**e**) early-onset Non-Hispanic White (NHW) treated with Bevacizumab; and (**f**) early-onset NHW without Bevacizumab.

Among EOCRC H/L patients treated with Bevacizumab, WNT status was not associated with survival (*p* = 0.60; Figure 2a), with closely overlapping curves and limited interpretability due to a very small, unaltered sample size. In contrast, among EOCRC H/L patients not treated with Bevacizumab, WNT alterations were associated with significantly improved survival (*p* = 0.014; Figure 2b), although estimates were limited by small numbers in the unaltered group.

For LOCRC H/L patients, WNT pathway status did not significantly impact survival regardless of Bevacizumab treatment (treated: *p* = 0.55, Figure 2c; untreated: *p* = 0.57, Figure 2d), with overlapping survival curves and increasing uncertainty at later follow-up.

Among EOCRC NHW patients treated with Bevacizumab, survival did not differ by WNT status (*p* = 0.88; Figure 2e). However, in EOCRC NHW patients not receiving Bevacizumab, WNT alterations were associated with significantly better survival (*p* = 0.023; Figure 2f).

Similarly, in LOCRC NHW patients treated with Bevacizumab, WNT status was not associated with survival (*p* = 0.38; Figure S2a), whereas in untreated LOCRC NHW patients, WNT pathway alterations were linked to improved overall survival (*p* = 0.0052; Figure S2b).

Overall, WNT pathway alterations were associated with improved survival primarily in untreated EOCRC and LOCRC NHW cohorts and untreated EOCRC H/L patients, while no survival differences were observed in Bevacizumab-treated groups, highlighting treatment context and sample size as key modifiers of observed effects.

### 2.7. AI-Enabled Integrative Analyses Across Bevacizumab-Treated Subgroups

To extend pathway-centric findings beyond the primary FOLFOX-treated AA cohort, we leveraged AI-HOPE and AI-HOPE-WNT to conduct integrative, treatment-focused analyses across ancestry- and age-defined colorectal cancer subgroups receiving Bevacizumab. These analyses were designed to assess whether WNT pathway status, specific WNT regulators, and broader clinical attributes exhibit consistent prognostic or molecular patterns under anti-angiogenic therapy.

Among early-onset H/L patients treated with Bevacizumab, AI-assisted cohort construction identified 26 WNT-altered and 4 WNT-unaltered cases. Survival analysis demonstrated overlapping Kaplan–Meier trajectories with no statistically significant difference in overall survival by WNT pathway status (Figure S3; log-rank *p* = 0.5966), reflecting both the rarity of this subgroup and the absence of a detectable prognostic effect in this context. Similarly, when restricting the analysis to Bevacizumab-treated patients harboring WNT alterations, ethnicity-stratified survival comparisons between H/L and NHW patients revealed no significant differences in overall survival (Figure S4; log-rank *p* = 0.4594), indicating comparable outcomes across ancestries within this treatment- and pathway-defined population.

Beyond survival, AI-enabled comparative genomics highlighted treatment- and ancestry-specific mutation patterns. In Bevacizumab-treated early-onset H/L versus NHW patients, RNF43 mutation prevalence was low and comparable between groups (Figure S5), with no significant difference detected by Fisher’s exact testing, suggesting similar disruption of this WNT regulator across ancestries under anti-angiogenic therapy. In contrast, within early-onset NHW patients, Bevacizumab exposure was associated with a marked reduction in TCF7L2 mutation frequency compared with untreated counterparts (Figure S6; *p* = 0.009; odds ratio = 0.498), implicating treatment-associated selection or interaction effects on specific WNT pathway components.

Finally, a broad AI-driven screen of categorical clinical and molecular attributes among Bevacizumab-treated H/L and NHW patients identified multiple variables significantly associated with ethnicity (Figure S7), including diagnostic and histologic descriptors, age at diagnosis, treatment-related variables, and selected pathway-linked gene alterations (e.g., AKT1 and SMAD3). Collectively, these analyses demonstrate how AI-enabled clinical informatics can efficiently interrogate heterogeneous datasets, delineate context-specific molecular patterns under targeted therapy, and generate testable hypotheses to inform disparity-aware precision oncology.

## 3. Discussion

This AI-driven, multi-cohort analysis delineates how WNT pathway dysregulation in colorectal cancer is shaped by age of onset, ancestry, and Bevacizumab exposure. Using AI-HOPE-WNT to harmonize clinical, genomic, and treatment data across 2517 H/L and NHW patients, three central findings emerge: EOCRC remains disproportionately prevalent in H/L populations; WNT disruption is nearly universal but modulated by age and therapy; and the prognostic impact of WNT alterations is context- and ancestry-dependent, particularly in untreated disease.

At the genomic level, APC truncation dominated across all strata, supporting WNT activation as a shared trunk event in CRC. However, secondary WNT regulators (RNF43, AXIN1/2, TCF7L2, AMER1, CTNNB1) varied by age, ancestry, and treatment, indicating multiple evolutionary routes to sustained WNT signaling. Bevacizumab exposure was consistently associated with lower mutation burden and TMB, alongside depletion of RNF43 and other non-canonical WNT alterations, suggesting therapy-driven selection or preferential benefit in tumors with simpler WNT architectures. Notably, RNF43 alterations were absent in treated H/L EOCRC and enriched in untreated tumors across ancestries, implicating specific WNT genotypes as potential modulators of anti-angiogenic response.

Age- and ancestry-stratified analyses further revealed that untreated EOCRC in both H/L and NHW patients showed stronger APC dependence, whereas untreated LOCRC, particularly in NHW patients, exhibited enrichment of non-canonical WNT regulators. Once Bevacizumab was introduced, WNT mutational landscapes converged across ancestries, with the exception of persistently higher chromosomal instability in treated H/L EOCRC, suggesting differential structural genomic vulnerability despite similar point mutation profiles.

Survival analyses demonstrated that WNT alterations were prognostically neutral in H/L patients regardless of treatment but associated with improved survival in untreated NHW EOCRC and LOCRC. This benefit disappeared with Bevacizumab exposure, implying that anti-angiogenic therapy may override baseline WNT-associated prognostic differences or select for more homogeneous post-treatment populations. These findings caution against population-agnostic interpretation of WNT biomarkers and highlight the need for ancestry-aware validation.

Methodologically, AI-HOPE-WNT enabled efficient, transparent interrogation of complex, heterogeneous datasets by supporting flexible cohort stratification and pathway-level inference without replacing conventional statistical rigor. This framework is particularly well suited for disparity-focused oncology research, where subgroup sizes are small and interactions between biology, treatment, and structural inequities are complex.

An important aspect of this study is the application of the conversational artificial intelligence frameworks AI-HOPE and AI-HOPE-WNT as scalable clinical–genomic interrogation platforms rather than predictive models. Although conventional bioinformatics pipelines remain essential for statistical testing and survival analyses, these approaches typically require extensive manual programming and repeated code modification to address evolving biological questions. In contrast, AI-HOPE and AI-HOPE-WNT enabled dynamic, natural language-driven exploration of integrated clinical and genomic datasets, allowing rapid construction of clinically matched cohorts defined simultaneously by age at diagnosis, ancestry, treatment exposure, and molecular characteristics. This capability facilitated real-time pathway-centric interrogation and iterative hypothesis generation across multiple clinically relevant contexts, substantially accelerating multidimensional data exploration. Importantly, all observations generated through the AI frameworks were subsequently validated using conventional statistical methodologies, ensuring analytical rigor and reproducibility. Thus, the primary contribution of these conversational AI systems lies in augmenting precision oncology research by enabling flexible, context-aware, and scalable interrogation of complex datasets that would otherwise require substantial bioinformatics expertise and manual analytical effort.

Despite these strengths, several limitations of the present study warrant consideration. Limitations include the retrospective nature of public datasets, incomplete treatment annotation, limited social determinants of health data, and small sample sizes in select subgroups. Prospective, longitudinal studies with functional validation will be required to confirm causal relationships between WNT genotypes, therapy response, and clonal evolution.

Additional considerations should be taken into account when interpreting the survival analyses presented in this study. First, survival comparisons were performed using Kaplan–Meier and log-rank methodologies without multivariable adjustment. Given the retrospective nature of the dataset and the likelihood of baseline clinical differences between Bevacizumab-treated and untreated patients, residual confounding cannot be excluded. Furthermore, the extensive stratification by age at diagnosis, ancestry, treatment exposure, and WNT pathway status resulted in relatively small subgroup sizes, limiting the feasibility and statistical robustness of multivariable Cox regression modeling. Consequently, the observed associations between WNT pathway alterations and clinical outcomes should be interpreted cautiously and viewed as exploratory and hypothesis-generating rather than definitive evidence of independent prognostic effects. Future studies involving larger, prospectively collected cohorts with detailed clinical annotation will be necessary to validate these findings and determine whether WNT pathway alterations retain prognostic significance after adjustment for established clinicopathologic variables.

Several additional limitations should be considered when interpreting the findings of this study. First, given the exploratory and hypothesis-generating nature of the analyses, formal correction for multiple comparisons was not applied. Consequently, the possibility of false-positive findings cannot be excluded, particularly considering the large number of subgroup analyses performed across ancestry, age at diagnosis, treatment exposure, and WNT pathway status. Second, extensive clinical and molecular stratification resulted in relatively small sample sizes in several subgroups, including some treatment- and ancestry-specific cohorts. These limited sample sizes reduce statistical power and may contribute to unstable effect estimates. Therefore, the associations identified in this study should be interpreted cautiously and viewed primarily as hypothesis-generating. Validation in larger, independent, and prospectively collected cohorts will be essential to confirm the robustness and generalizability of these observations, particularly in historically understudied Hispanic/Latino populations [41,42,43].

An additional challenge of this study is the exploratory nature of the analyses, which is largely driven by the limited availability of comprehensive, clinically annotated genomic datasets from H/L patients with colorectal cancer. H/L populations remain substantially underrepresented in large-scale cancer genomics initiatives, restricting the ability to perform adequately powered ancestry-specific analyses and independent validations. Through multiple initiatives, different efforts have begun establishing some of the first comprehensive multi-omics colorectal cancer cohorts enriched for Hispanic/Latino patients, integrating genomic, transcriptomic, ancestry, treatment, and clinical outcome data [43]. As these resources continue to expand, future studies will incorporate additional H/L datasets to validate and refine the ancestry-specific associations identified herein, ultimately improving the generalizability and translational relevance of precision oncology strategies for this underserved population.

## 4. Materials and Methods

### 4.1. Data Sources and Cohort Assembly

Clinical, genomic, and treatment data for CRC patients were aggregated from multiple publicly accessible datasets hosted within cBioPortal for Cancer Genomics. Datasets were selected based on their availability of comprehensive clinical annotations, chemotherapeutic exposure fields, and high-quality somatic mutation profiles. The final analytic cohort included CRC cases from: TCGA Colorectal Adenocarcinoma (PanCancer Atlas), MSK-CHORD Colorectal Cancer Dataset, and AACR GENIE Biopharma Collaborative (BPC) CRC Cohort.

Only primary adenocarcinomas of the colon and rectum with matched clinical and genomic data were included. For patients with multiple tumor samples, a single primary tumor specimen per individual was retained to avoid duplication bias.

Race and ethnicity were extracted from structured clinical annotations. Individuals labeled as “Hispanic or Latino,” “Hispanic, NOS,” “Spanish, NOS,” or “Latino, NOS” were categorized as Hispanic/Latino (H/L). NHW status was defined as “White” or “Non-Hispanic White.” Age at diagnosis was used to classify patients as EOCRC (<50 years) or LOCRC (≥50 years). Bevacizumab exposure was identified through anticancer drug administration fields and verified manually through treatment timelines when available.

Across all datasets, patients were stratified into subgroups defined by age (EOCRC vs. LOCRC), ancestry (H/L vs. NHW), presence or absence of bevacizumab treatment, and WNT pathway alteration status.

### 4.2. Genomic Annotation and WNT Pathway Classification

Somatic mutation data were retrieved from cBioPortal’s standardized MAF files and converted into gene-level alteration matrices. Mutations were limited to nonsynonymous changes, including missense, nonsense, frameshift insertions/deletions, splice site variants, and translation start site alterations.

WNT pathway genes were curated from established CRC literature and included core members and regulators such as APC, CTNNB1, RNF43, AXIN1, AXIN2, TCF7L2, AMER1, and others. A case was classified as “WNT-altered” if at least one qualifying nonsynonymous mutation was detected in any pathway gene. Gene-level and pathway-level mutation rates were quantified across all demographic and treatment subgroups. Co-alteration patterns, including concurrent mutations within or across pathways, were examined to identify biologically relevant patterns associated with treatment exposure.

### 4.3. Statistical and Survival Analyses

Mutation frequencies between groups were compared using chi-square tests; Fisher’s exact tests were applied when expected cell counts were <5. Comparisons included: EOCRC vs. LOCRC, H/L vs. NHW, bevacizumab-exposed vs. non-exposed, and WNT-altered vs. WNT-wild-type tumors.

Kaplan–Meier survival analyses were performed to assess associations between WNT alterations and overall survival across age, ancestry, and treatment subgroups. Log-rank tests were used to compare survival curves, and median survival with 95% confidence intervals (CIs) was reported. Subgroup analyses were conducted to evaluate whether prognostic effects of WNT alterations differed by bevacizumab exposure.

All statistical analyses were conducted using R (v4.3) and Python (v3.11). A two-sided *p*-value < 0.05 was considered statistically significant.

### 4.4. AI-Enabled Clinical–Genomic Integration and Pathway-Oriented Analysis

To support systematic integration of heterogeneous clinical, genomic, and treatment data, we employed AI-HOPE [38] and its pathway-specific extension AI-HOPE-WNT [39], artificial intelligence-based clinical informatics frameworks developed for precision oncology research [40,44]. These platforms were used to harmonize structured clinical annotations, somatic mutation profiles, and therapeutic exposure variables into unified analytical representations suitable for pathway-level investigation.

The AI frameworks facilitated dynamic cohort definition and multivariable stratification across age at diagnosis, ancestry, Bevacizumab exposure, and WNT pathway alteration status. Rather than performing statistical inference directly, the AI systems were used to organize, summarize, and prioritize clinically interpretable genomic features and subgroup comparisons for downstream hypothesis testing. This included automated aggregation of gene- and pathway-level mutation frequencies, alignment of treatment timelines with molecular profiles, and identification of ancestry- and treatment-dependent patterns warranting formal statistical evaluation.

By reducing manual data manipulation and enforcing consistent analytic logic across cohorts, the AI-assisted workflow improved the reproducibility and scalability of the analyses. Importantly, all survival modeling and statistical testing were conducted independently using conventional biostatistical methods, ensuring that AI outputs served as an informatics support layer rather than a replacement for established statistical inference. This hybrid approach enabled efficient, transparent exploration of complex clinical–genomic interactions while maintaining methodological rigor.

## 5. Conclusions

WNT pathway disruption is a universal feature of colorectal cancer, but its architecture and clinical significance vary by ancestry, age of onset, and Bevacizumab exposure. While APC inactivation defines a shared oncogenic core, secondary WNT regulators exhibit treatment- and ancestry-specific patterns with distinct prognostic implications. The differential survival associations observed across populations underscore the necessity of integrating ancestry-aware genomics with detailed treatment context to advance equitable precision oncology, particularly for populations disproportionately affected by early-onset CRC.

## Figures and Tables

**Table 1:** Overview of clinical features, demographics, and treatment exposure in H/L and NHW colorectal cancer cohorts.

Clinical Feature	H/L Cohort n (%)	NHW Cohort n (%)
Age Onset & Treatment		
Early-Onset (<50) Treated with Bevacizumab	30 (10.9%)	172 (7.0%)
Late-Onset (≥50) Treated with Bevacizumab	43 (15.6%)	371 (15.2%)
Early-Onset (<50) Not Treated with Bevacizumab	91 (33.1%)	569 (23.3%)
Late-Onset (≥50) Not Treated with Bevacizumab	111 (40.4%)	1330 (54.5%)
Cancer Type		
Colon Adenocarcinoma	8 (2.9%)	1402 (57.4%)
Rectal Adenocarcinoma	1 (0.4%)	711 (29.1%)
Colorectal Adenocarcinoma	266 (96.7%)	329 (13.5%)
Sex		
Male	158 (57.5%)	1339 (54.8%)
Female	117 (42.5%)	1103 (45.2%)
Sample Type		
Primary Tumor	275 (100.0%)	2442 (100.0%)
Stage (Highest Recorded)		
Stage 1-3	162 (58.9%)	1499 (61.4%)
Stage 4	113 (41.1%)	943 (38.6%)
MSI Type		
Stable	213 (77.5%)	2082 (85.3%)
Instable	33 (12.0%)	271 (11.1%)
Indeterminate	10 (3.6%)	60 (2.5%)
NA	19 (6.9%)	29 (1.2%)
Ethnicity		
Spanish NOS; Hispanic NOS, Latino NOS	248 (90.2%)	0 (0.0%)
Mexican (includes Chicano)	19 (6.9%)	0 (0.0%)
Hispanic or Latino	4 (1.5%)	0 (0.0%)
Spanish surname only	4 (1.5%)	0 (0.0%)
Non-Spanish; Non-Hispanic	0 (0.0%)	2442 (100.0%)

**Table 2:** Clinical and genomic comparisons between early-onset and late-onset colorectal cancer (CRC) cohorts. This table presents key molecular distinctions related to WNT pathway dysregulation and mutation burden across major subgroups: (a) Early-Onset CRC (EOCRC) versus Late-Onset CRC (LOCRC) among Hispanic/Latino (H/L) patients; (b) EOCRC versus LOCRC among Non-Hispanic White (NHW) patients. Each analysis includes the prevalence of specific WNT pathway gene alterations, organized by ethnicity and age category.

**(a)**						
**Clinical Feature**	**Early-Onset Hispanic/Latino** **Treated with Bevacizumab** ***n* (%)**	**Early-Onset Hispanic/Latino** **Not Treated with Bevacizumab** ***n* (%)**	***p*-Value**	**Late-Onset Hispanic/Latino** **Treated with Bevacizumab** ***n* (%)**	**Late-Onset Hispanic/Latino** **Not Treated with Bevacizumab** ***n* (%)**	***p*-Value**
RNF43 Mutation						
Present	0 (0.0%)	13 (14.3%)	0.03657	1 (2.3%)	20 (18.0%)	0.008617
Absent	30 (100.0%)	78 (85.7%)		42 (97.7%)	91 (82.0%)	
**(b)**						
**Clinical Feature**	**Early-Onset NHW** **Treated with Bevacizumab** ***n* (%)**	**Early-Onset NHW** **Not Treated with Bevacizumab** ***n* (%)**	***p*-Value**	**Late-Onset NHW** **Treated with Bevacizumab** ***n* (%)**	**Late-Onset NHW** **Not Treated with Bevacizumab** ***n* (%)**	***p*-Value**
AMER1 Mutation						
Present	8 (4.7%)	45 (7.9%)	0.1992	22 (5.9%)	146 (11.0%)	0.005384
Absent	164 (95.3%)	524 (92.1%)		349 (94.1%)	1184 (89.0%)	
AXIN1 Mutation						
Present	0 (0.0%)	15 (2.6%)	0.02845	3 (0.8%)	63 (4.7%)	1.84 × 10^−4^
Absent	172 (100.0%)	554 (97.4%)		368 (99.2%)	1267 (95.3%)	
AXIN2 Mutation						
Present	2 (1.2%)	29 (5.1%)	0.02724	14 (3.8%)	110 (8.3%)	0.004607
Absent	170 (98.8%)	540 (94.9%)		357 (96.2%)	1220 (91.7%)	
RNF43 Mutation						
Present	3 (1.7%)	44 (7.7%)	0.003625	18 (4.9%)	183 (13.8%)	4.05 × 10^−6^
Absent	169 (98.3%)	525 (92.3%)		353 (95.1%)	1147 (86.2%)	
TCF7L2 Mutation						
Present	20 (11.6%)	119 (20.9%)	0.008735	44 (11.9%)	225 (16.9%)	0.01396
Absent	152 (88.4%)	450 (79.1%)		327 (88.1%)	1105 (83.1%)	

## Data Availability

All datasets analyzed are publicly available via cBioPortal (accessed 11 November 2025) and the AACR Project GENIE platform (accessed 11 November 2025). Code and analytical resources supporting this work are available through the GitHub repository https://github.com/Velazquez-Villarreal-Lab/AI-HOPE-WNT (accessed 11 November 2025) to ensure reproducibility and transparency. Additional materials may be provided upon reasonable request.

## Supplementary Materials

The following supporting information can be downloaded at https://www.mdpi.com/article/10.3390/ijms27146195/s1.
