# Supplementary material for: Artificial Intelligence-Enabled Analysis of WNT Pathway Dysregulation in Bevacizumab-Treated Early-Onset Colorectal Cancer
Source: Int J Mol Sci. 2026 Jul 11;27(14):6195. doi: 10.3390/ijms27146195 (PMC13410101; doi:10.3390/ijms27146195)
Supplement: Supplementary file 1 [file ijms-27-06195-s001.zip › ijms-4351336-supplementary.pdf]

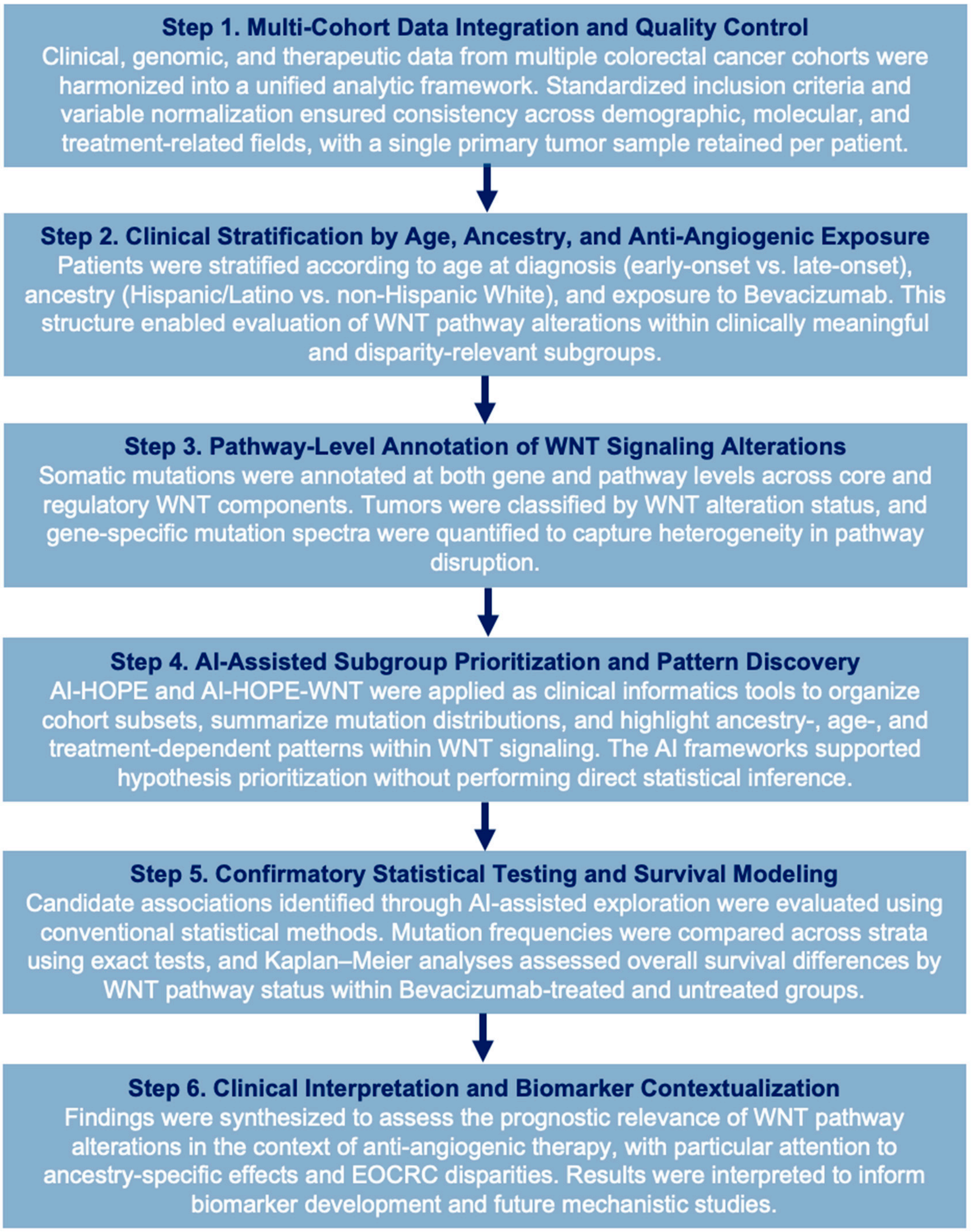

Figure S1. Analytical Framework for Evaluating WNT Pathway Dysregulation in Bevacizumab-Treated Colorectal Cancer. The figure illustrates the sequential integration of multi-cohort clinical, genomic, and treatment data followed by stratification according to age at diagnosis, ancestry, and exposure to anti-angiogenic therapy. WNT pathway alterations were annotated at the gene and pathway levels across core signaling components. Artificial intelligence-enabled informatics tools were applied to support cohort organization, subgroup prioritization, and identification of treatment- and ancestry-dependent molecular patterns. Candidate associations were subsequently evaluated using conventional statistical methods, including group-wise comparisons of mutation frequencies and time-to-event analyses of overall survival. This framework enabled systematic assessment of the prognostic relevance of WNT pathway dysregulation within clinically and demographically defined subpopulations.

Table S1. Clinical and genomic comparisons between early-onset and late-onset colorectal cancer (CRC) cohorts. This table presents key clinical and molecular distinctions related to WNT pathway dysregulation and mutation burden across major subgroups: (a) Early-Onset CRC (EOCRC) versus Late-Onset CRC (LOCRC) among Hispanic/Latino (H/L) patients; (b) EOCRC versus LOCRC among Non-Hispanic White (NHW) patients; and (c) cross-ethnic comparisons of EOCRC between H/L and NHW groups. Each analysis includes age at diagnosis, total mutational load, and prevalence of specific WNT pathway gene alterations, organized by ethnicity and age category.

A)

Clinical Feature	Early-Onset Hispanic/Latino Treated with Bevacizumab n (%)	Early-Onset Hispanic/Latino Not Treated with Bevacizumab n (%)	p-value	Late-Onset Hispanic/Latino Treated with Bevacizumab n (%)	Late-Onset Hispanic/Latino Not Treated with Bevacizumab n (%)	p-value
Median Diagnosis Age (IQR)	43 (40-45)	41 (36-46)	0.1229	60 (55-64)	61 (55-71)	0.105
Median Mutation Count	6 (4-7)	8 (5-13)	**0.003203**	8 (6.5-9)	8 (6-11)	0.6157
Median TMB (IQR)	4.9 (3.7-6.3) [NA=3]	6.1 (4.1-10.7) [NA=6]	**0.01245**	6.7 (5.2-7.4) [NA=5]	6.6 (4.9-9.5) [NA=4]	0.7039
Median FGA	0.24 (0.15-0.32) [NA=1]	0.17 (0.01-0.27) [NA=4]	**0.009103**	0.15 (0.04-0.25) [NA=4]	0.15 (0.03-0.26) [NA=5]	0.8201

B)

Clinical Feature	Early-Onset NHW Treated with Bevacizumab n (%)	Early-Onset NHW Not Treated with Bevacizumab n (%)	p-value	Late-Onset NHW Treated with Bevacizumab n (%)	Late-Onset NHW Not Treated with Bevacizumab n (%)	p-value
Median Diagnosis Age (IQR)	43 (37-47)	44 (38-47)	0.8206	62 (56-69)	65 (57-73)	**0.00105**
Median Mutation Count	6 (4-7.5) [NA=1]	7 (5-9) [NA=3]	**0.001662**	7 (5-9) [NA=2]	8 (5-11) [NA=5]	**1.84E-06**
Median TMB (IQR)	5.5 (4.1-6.9)	5.7 (4.1-7.8)	**0.04211**	6.1 (4.3-7.8)	6.6 (4.4-9.7)	**0.0001393**
Median FGA	0.17 (0.06-0.25) [NA=1]	0.15 (0.04-0.24) [NA=6]	0.1289	0.18 (0.07-0.29) [NA=2]	0.15 (0.05-0.27) [NA=13]	**0.00713**

C)

Clinical Feature	Early-Onset Hispanic/Latino Treated with Bevacizumab n (%)	Early-Onset NHW Treated with Bevacizumab n (%)	p-value	Early-Onset Hispanic/Latino Not Treated with Bevacizumab n (%)	Early-Onset NHW Not Treated with Bevacizumab n (%)	p-value
Median Diagnosis Age (IQR)	43 (40-45)	43 (37-47)	0.7671	41 (36-46)	44 (38-47)	**0.001968**
Median Mutation Count	6 (4-7)	6 (4-7.5) [NA=1]	0.4613	8 (5-13)	7 (5-9) [NA=3]	0.0562
Median TMB (IQR)	4.9 (3.7-6.3) [NA=3]	5.5 (4.1-6.9)	0.2353	6.1 (4.1-10.7) [NA=6]	5.7 (4.1-7.8)	0.1636
Median FGA	0.24 (0.15-0.32) [NA=1]	0.17 (0.06-0.25) [NA=1]	0.008669	0.17 (0.01-0.27) [NA=4]	0.15 (0.04-0.24) [NA=6]	0.7813

Table S2. WNT signaling alterations across Hispanic/Latino CRC subgroups classified by age and Bevacizumab therapy. This table presents mutation frequencies for principal WNT pathway genes in H/L CRC patients and includes: (3a) age-of-onset differences within the H/L cohort, (3b) treatment-based variation across EOCRC and LOCRC, (3c) early-onset comparisons between H/L and NHW patients, and (3d) late-onset comparisons by ancestry and treatment status.

(a)

Pathway Alterations	Early-Onset Hispanic/Latino Treated with Bevacizumab n (%)	Early-Onset Hispanic/Latino Not Treated with Bevacizumab n (%)	p-value	Late-Onset Hispanic/Latino Treated with Bevacizumab n (%)	Late-Onset Hispanic/Latino Not Treated with Bevacizumab n (%)	p-value
WNT Alterations Present	26 (86.7%)	83 (91.2%)	0.4892	36 (83.7%)	94 (84.7%)	1
WNT Alterations Absent	4 (13.3%)	8 (8.8%)		7 (16.3%)	17 (15.3%)	

(b)

Pathway Alterations	Early-Onset NHW Treated with Bevacizumab n (%)	Early-Onset NHW Not Treated with Bevacizumab n (%)	p-value	Late-Onset NHW Treated with Bevacizumab n (%)	Late-Onset NHW Not Treated with Bevacizumab n (%)	p-value
WNT Alterations Present	141 (82.0%)	499 (87.7%)	0.07355	330 (88.9%)	1177 (88.5%)	0.8807
WNT Alterations Absent	31 (18.0%)	70 (12.3%)		41 (11.1%)	153 (11.5%)	

(c)

Pathway Alterations	Early-Onset Hispanic/Latino Treated with Bevacizumab n (%)	Early-Onset NHW Treated with Bevacizumab n (%)	p-value	Early-Onset Hispanic/Latino Not Treated with Bevacizumab n (%)	Early-Onset NHW Not Treated with Bevacizumab n (%)	p-value
WNT Alterations Present	26 (86.7%)	141 (82.0%)	0.7936	83 (91.2%)	499 (87.7%)	0.4304
WNT Alterations Absent	4 (13.3%)	31 (18.0%)		8 (8.8%)	70 (12.3%)	

(d)

Pathway Alterations	Late-Onset Hispanic/Latino Treated with Bevacizumab n (%)	Late-Onset NHW Treated with Bevacizumab n (%)	p-value	Late-Onset Hispanic/Latino Not Treated with Bevacizumab n (%)	Late-Onset NHW Not Treated with Bevacizumab n (%)	p-value
WNT Alterations Present	36 (83.7%)	330 (88.9%)	0.446	94 (84.7%)	1177 (88.5%)	0.297
WNT Alterations Absent	7 (16.3%)	41 (11.1%)		17 (15.3%)	153 (11.5%)	

Table S3. - EO HL Treated with Bevacizumab vs EO HL Not Treated with Bevacizumab.

WNT Pathway			
**Gene**	**Early-Onset Hispanic/Latino Treated with Bevacizumab n (%)**	**Early-Onset Hispanic/Latino Not Treated with Bevacizumab n (%)**	**p-value**
**AMER1 Mutation**			
Present	2 (6.7%)	10 (11.0%)	0.7281
Absent	28 (93.3%)	81 (89.0%)	
**APC Mutation**			
Present	24 (80.0%)	76 (83.5%)	0.8704.
Absent	6 (20.0%)	15 (16.5%)	
**AXIN1 Mutation**			
Present	0 (0.0%)	2 (2.2%)	1
Absent	30 (100.0%)	89 (97.8%)	
**AXIN2 Mutation**			
Present	1 (3.3%)	4 (4.4%)	1
Absent	29 (96.7%)	87 (95.6%)	
**CTNNB1 Mutation**			
Present	2 (6.7%)	9 (9.9%)	0.7297
Absent	28 (93.3%)	82 (90.1%)	
**GSK3B Mutation**			
Present	0 (0.0%)	1 (1.1%)	1
Absent	30 (100.0%)	90 (98.9%)	
**RNF43 Mutation**			
Present	0 (0.0%)	13 (14.3%)	**0.03657**
Absent	30 (100.0%)	78 (85.7%)	
**TCF7L2 Mutation**			
Present	3 (10.0%)	18 (19.8%)	0.2759
Absent	27 (90.0%)	73 (80.2%)	
**TLE1 Mutation**			
Present	0 (0.0%)	1 (1.1%)	1
Absent	30 (100.0%)	90 (98.9%)	
**TLE2 Mutation**			
Present	0 (0.0%)	1 (1.1%)	1
Absent	30 (100.0%)	90 (98.9%)	

Table S4. LO HL Treated with Bevacizumab v LO HL Not Treated with Bevacizumab.

WNT Pathway			
**Gene**	**Late-Onset Hispanic/Latino Treated with Bevacizumab n (%)**	**Late-Onset Hispanic/Latino Not Treated with Bevacizumab n (%)**	**p-value**
**AMER1 Mutation**			
Present	5 (11.6%)	7 (6.3%)	0.4412
Absent	38 (88.4%)	104 (93.7%)	
**APC Mutation**			
Present	34 (79.1%)	73 (65.8%)	0.1575
Absent	9 (20.9%)	38 (34.2%)	
**AXIN1 Mutation**			
Present	0 (0.0%)	3 (2.7%)	0.5602
Absent	43 (100.0%)	108 (97.3%)	
**AXIN2 Mutation**			
Present	0 (0.0%)	8 (7.2%)	0.1067
Absent	43 (100.0%)	103 (92.8%)	
**CTNNB1 Mutation**			
Present	3 (7.0%)	7 (6.3%)	1
Absent	40 (93.0%)	104 (93.7%)	
**GSK3B Mutation**			
Present	0 (0.0%)	1 (0.9%)	1
Absent	43 (100.0%)	110 (99.1%)	
**RNF43 Mutation**			
Present	1 (2.3%)	20 (18.0%)	**0.008617**
Absent	42 (97.7%)	91 (82.0%)	
**TCF7L2 Mutation**			
Present	5 (11.6%)	15 (13.5%)	0.964
Absent	38 (88.4%)	96 (86.5%)	
**TLE1 Mutation**			
Present	0 (0.0%)	0 (0.0%)	1
Absent	43 (100.0%)	111 (100.0%)	
**TLE2 Mutation**			
Present	0 (0.0%)	0 (0.0%)	1
Absent	43 (100.0%)	111 (100.0%)	

Table S5. EO HL Treated with Bevacizumab v LO HL Treated with Bevacizumab.

WNT Pathway			
**Gene**	**Early-Onset Hispanic/Latino Treated with Bevacizumab n (%)**	**Late-Onset Hispanic/Latino Treated with Bevacizumab n (%)**	**p-value**
**AMER1 Mutation**			
Present	2 (6.7%)	5 (11.6%)	0.6925
Absent	28 (93.3%)	38 (88.4%)	
**APC Mutation**			
Present	24 (80.0%)	34 (79.1%)	1
Absent	6 (20.0%)	9 (20.9%)	
**AXIN1 Mutation**			
Present	0 (0.0%)	0 (0.0%)	1
Absent	30 (100.0%)	43 (100.0%)	
**AXIN2 Mutation**			
Present	1 (3.3%)	0 (0.0%)	0.411
Absent	29 (96.7%)	43 (100.0%)	
**CTNNB1 Mutation**			
Present	2 (6.7%)	3 (7.0%)	1
Absent	28 (93.3%)	40 (93.0%)	
**GSK3B Mutation**			
Present	0 (0.0%)	0 (0.0%)	1
Absent	30 (100.0%)	43 (100.0%)	
**RNF43 Mutation**			
Present	0 (0.0%)	1 (2.3%)	1
Absent	30 (100.0%)	42 (97.7%)	
**TCF7L2 Mutation**			
Present	3 (10.0%)	5 (11.6%)	1
Absent	27 (90.0%)	38 (88.4%)	
**TLE1 Mutation**			
Present	0 (0.0%)	0 (0.0%)	1
Absent	30 (100.0%)	43 (100.0%)	
**TLE2 Mutation**			
Present	0 (0.0%)	0 (0.0%)	1
Absent	30 (100.0%)	43 (100.0%)	

Table S6. EO HL Not Treated with Bevacizumab v LO HL Not Treated with Bevacizumab.

WNT Pathway			
Gene	Early-Onset Hispanic/Latino Not Treated with Bevacizumab n (%)	Late-Onset Hispanic/Latino Not Treated with Bevacizumab n (%)	p-value
**AMER1 Mutation**			
Present	10 (11.0%)	7 (6.3%)	0.3482
Absent	81 (89.0%)	104 (93.7%)	
**APC Mutation**			
Present	76 (83.5%)	73 (65.8%)	0.007091
Absent	15 (16.5%)	38 (34.2%)	
**AXIN1 Mutation**			
Present	2 (2.2%)	3 (2.7%)	1
Absent	89 (97.8%)	108 (97.3%)	
**AXIN2 Mutation**			
Present	4 (4.4%)	8 (7.2%)	0.5527
Absent	87 (95.6%)	103 (92.8%)	
**CTNNB1 Mutation**			
Present	9 (9.9%)	7 (6.3%)	0.4987
Absent	82 (90.1%)	104 (93.7%)	
**GSK3B Mutation**			
Present	1 (1.1%)	1 (0.9%)	1
Absent	90 (98.9%)	110 (99.1%)	
**RNF43 Mutation**			
Present	13 (14.3%)	20 (18.0%)	0.6012
Absent	78 (85.7%)	91 (82.0%)	
**TCF7L2 Mutation**			
Present	18 (19.8%)	15 (13.5%)	0.3137
Absent	73 (80.2%)	96 (86.5%)	
**TLE1 Mutation**			
Present	1 (1.1%)	0 (0.0%)	0.4505
Absent	90 (98.9%)	111 (100.0%)	
**TLE2 Mutation**			
Present	1 (1.1%)	0 (0.0%)	0.4505
Absent	90 (98.9%)	111 (100.0%)	

Table S7. EO NHW Treated with Bevacizumab v EO NHW Not Treated with Bevacizumab.

WNT Pathway			
**Gene**	**Early-Onset NHW Treated with Bevacizumab n (%)**	**Early-Onset NHW Not Treated with Bevacizumab n (%)**	**p-value**
**AMER1 Mutation**			
Present	8 (4.7%)	45 (7.9%)	0.1992
Absent	164 (95.3%)	524 (92.1%)	
**APC Mutation**			
Present	129 (75.0%)	460 (80.8%)	0.1199
Absent	43 (25.0%)	109 (19.2%)	
**AXIN1 Mutation**			
Present	0 (0.0%)	15 (2.6%)	**0.02845**
Absent	172 (100.0%)	554 (97.4%)	
**AXIN2 Mutation**			
Present	2 (1.2%)	29 (5.1%)	**0.02724**
Absent	170 (98.8%)	540 (94.9%)	
**CTNNB1 Mutation**			
Present	10 (5.8%)	34 (6.0%)	1
Absent	162 (94.2%)	535 (94.0%)	
**GSK3B Mutation**			
Present	1 (0.6%)	6 (1.1%)	1
Absent	171 (99.4%)	563 (98.9%)	
**RNF43 Mutation**			
Present	3 (1.7%)	44 (7.7%)	**0.003625**
Absent	169 (98.3%)	525 (92.3%)	
**TCF7L2 Mutation**			
Present	20 (11.6%)	119 (20.9%)	**0.008735**
Absent	152 (88.4%)	450 (79.1%)	
**TLE1 Mutation**			
Present	0 (0.0%)	0 (0.0%)	1
Absent	172 (100.0%)	569 (100.0%)	
**TLE2 Mutation**			
Present	0 (0.0%)	0 (0.0%)	1
Absent	172 (100.0%)	569 (100.0%)	

Table S8. LO NHW Treated with Bevacizumab v LO NHW Not Treated with Bevacizumab.

WNT Pathway			
**Gene**	**Late-Onset NHW Treated with Bevacizumab n (%)**	**Late-Onset NHW Not Treated with Bevacizumab n (%)**	**p-value**
**AMER1 Mutation**			
Present	22 (5.9%)	146 (11.0%)	**0.005384**
Absent	349 (94.1%)	1184 (89.0%)	
**APC Mutation**			
Present	297 (80.1%)	999 (75.1%)	0.05653
Absent	74 (19.9%)	331 (24.9%)	
**AXIN1 Mutation**			
Present	3 (0.8%)	63 (4.7%)	**0.0001839**
Absent	368 (99.2%)	1267 (95.3%)	
**AXIN2 Mutation**			
Present	14 (3.8%)	110 (8.3%)	**0.004607**
Absent	357 (96.2%)	1220 (91.7%)	
**CTNNB1 Mutation**			
Present	22 (5.9%)	93 (7.0%)	0.5459
Absent	349 (94.1%)	1237 (93.0%)	
**GSK3B Mutation**			
Present	1 (0.3%)	17 (1.3%)	0.1465
Absent	370 (99.7%)	1313 (98.7%)	
**RNF43 Mutation**			
Present	18 (4.9%)	183 (13.8%)	**4.05E-06**
Absent	353 (95.1%)	1147 (86.2%)	
**TCF7L2 Mutation**			
Present	44 (11.9%)	225 (16.9%)	**0.01396**
Absent	327 (88.1%)	1105 (83.1%)	
**TLE1 Mutation**			
Present	0 (0.0%)	0 (0.0%)	**1**
Absent	371 (100.0%)	1330 (100.0%)	
**TLE2 Mutation**			
Present	0 (0.0%)	0 (0.0%)	**1**
Absent	371 (100.0%)	1330 (100.0%)	

Table S9. EO NHW Treated with Bevacizumab v LO NHW Treated with Bevacizumab.

WNT Pathway			
**Gene**	**Early-Onset NHW Treated with Bevacizumab n (%)**	**Late-Onset NHW Treated with Bevacizumab n (%)**	**p-value**
**AMER1 Mutation**			
Present	8 (4.7%)	22 (5.9%)	0.6856
Absent	164 (95.3%)	349 (94.1%)	
**APC Mutation**			
Present	129 (75.0%)	297 (80.1%)	0.2223
Absent	43 (25.0%)	74 (19.9%)	
**AXIN1 Mutation**			
Present	0 (0.0%)	3 (0.8%)	0.5551
Absent	172 (100.0%)	368 (99.2%)	
**AXIN2 Mutation**			
Present	2 (1.2%)	14 (3.8%)	0.1083
Absent	170 (98.8%)	357 (96.2%)	
**CTNNB1 Mutation**			
Present	10 (5.8%)	22 (5.9%)	1
Absent	162 (94.2%)	349 (94.1%)	
**GSK3B Mutation**			
Present	1 (0.6%)	1 (0.3%)	0.5336
Absent	171 (99.4%)	370 (99.7%)	
**RNF43 Mutation**			
Present	3 (1.7%)	18 (4.9%)	0.09578
Absent	169 (98.3%)	353 (95.1%)	
**TCF7L2 Mutation**			
Present	20 (11.6%)	44 (11.9%)	1
Absent	152 (88.4%)	327 (88.1%)	
**TLE1 Mutation**			
Present	0 (0.0%)	0 (0.0%)	1
Absent	172 (100.0%)	371 (100.0%)	
**TLE2 Mutation**			
Present	0 (0.0%)	0 (0.0%)	1
Absent	172 (100.0%)	371 (100.0%)	

Table S10. EO NHW Not Treated with Bevacizumab v LO NHW Not Treated with Bevacizumab.

WNT Pathway			
**Gene**	**Early-Onset NHW Not Treated with Bevacizumab n (%)**	**Late-Onset NHW Not Treated with Bevacizumab n (%)**	**p-value**
**AMER1 Mutation**			
Present	45 (7.9%)	146 (11.0%)	0.05075
Absent	524 (92.1%)	1184 (89.0%)	
**APC Mutation**			
Present	460 (80.8%)	999 (75.1%)	**0.007999**
Absent	109 (19.2%)	331 (24.9%)	
**AXIN1 Mutation**			
Present	15 (2.6%)	63 (4.7%)	**0.04695**
Absent	554 (97.4%)	1267 (95.3%)	
**AXIN2 Mutation**			
Present	29 (5.1%)	110 (8.3%)	**0.01946**
Absent	540 (94.9%)	1220 (91.7%)	
**CTNNB1 Mutation**			
Present	34 (6.0%)	93 (7.0%)	0.4762
Absent	535 (94.0%)	1237 (93.0%)	
**GSK3B Mutation**			
Present	6 (1.1%)	17 (1.3%)	0.8577
Absent	563 (98.9%)	1313 (98.7%)	
**RNF43 Mutation**			
Present	44 (7.7%)	183 (13.8%)	**0.0002822**
Absent	525 (92.3%)	1147 (86.2%)	
**TCF7L2 Mutation**			
Present	119 (20.9%)	225 (16.9%)	**0.0448**
Absent	450 (79.1%)	1105 (83.1%)	
**TLE1 Mutation**			
Present	0 (0.0%)	0 (0.0%)	1
Absent	569 (100.0%)	1330 (100.0%)	
**TLE2 Mutation**			
Present	0 (0.0%)	0 (0.0%)	1
Absent	569 (100.0%)	1330 (100.0%)	

Table S11. EO HL Treated with Bevacizumab v EO NHW Treated with Bevacizumab.

WNT Pathway			
**Gene**	**Early-Onset Hispanic/Latino Treated with Bevacizumab n (%)**	**Early-Onset NHW Treated with Bevacizumab n (%)**	**p-value**
**AMER1 Mutation**			
Present	2 (6.7%)	8 (4.7%)	0.6458
Absent	28 (93.3%)	164 (95.3%)	
**APC Mutation**			
Present	24 (80.0%)	129 (75.0%)	0.7198
Absent	6 (20.0%)	43 (25.0%)	
**AXIN1 Mutation**			
Present	0 (0.0%)	0 (0.0%)	1
Absent	30 (100.0%)	172 (100.0%)	
**AXIN2 Mutation**			
Present	1 (3.3%)	2 (1.2%)	0.3843
Absent	29 (96.7%)	170 (98.8%)	
**CTNNB1 Mutation**			
Present	2 (6.7%)	10 (5.8%)	0.694
Absent	28 (93.3%)	162 (94.2%)	
**GSK3B Mutation**			
Present	0 (0.0%)	1 (0.6%)	1
Absent	30 (100.0%)	171 (99.4%)	
**RNF43 Mutation**			
Present	0 (0.0%)	3 (1.7%)	1
Absent	30 (100.0%)	169 (98.3%)	
**TCF7L2 Mutation**			
Present	3 (10.0%)	20 (11.6%)	1
Absent	27 (90.0%)	152 (88.4%)	
**TLE1 Mutation**			
Present	0 (0.0%)	0 (0.0%)	1
Absent	30 (100.0%)	172 (100.0%)	
**TLE2 Mutation**			
Present	0 (0.0%)	0 (0.0%)	1
Absent	30 (100.0%)	172 (100.0%)	

Table S12. EO HL Not Treated with Bevacizumab v EO NHW Not Treated with Bevacizumab.

WNT Pathway			
**Gene**	**Early-Onset Hispanic/Latino Not Treated with Bevacizumab n (%)**	**Early-Onset NHW Not Treated with Bevacizumab n (%)**	**p-value**
**AMER1 Mutation**			
Present	10 (11.0%)	45 (7.9%)	0.4337
Absent	81 (89.0%)	524 (92.1%)	
**APC Mutation**			
Present	76 (83.5%)	460 (80.8%)	0.6444
Absent	15 (16.5%)	109 (19.2%)	
**AXIN1 Mutation**			
Present	2 (2.2%)	15 (2.6%)	1
Absent	89 (97.8%)	554 (97.4%)	
**AXIN2 Mutation**			
Present	4 (4.4%)	29 (5.1%)	1
Absent	87 (95.6%)	540 (94.9%)	
**CTNNB1 Mutation**			
Present	9 (9.9%)	34 (6.0%)	0.2395
Absent	82 (90.1%)	535 (94.0%)	
**GSK3B Mutation**			
Present	1 (1.1%)	6 (1.1%)	1
Absent	90 (98.9%)	563 (98.9%)	
**RNF43 Mutation**			
Present	13 (14.3%)	44 (7.7%)	0.06214
Absent	78 (85.7%)	525 (92.3%)	
**TCF7L2 Mutation**			
Present	18 (19.8%)	119 (20.9%)	0.9137
Absent	73 (80.2%)	450 (79.1%)	
**TLE1 Mutation**			
Present	1 (1.1%)	0 (0.0%)	0.1379
Absent	90 (98.9%)	569 (100.0%)	
**TLE2 Mutation**			
Present	1 (1.1%)	0 (0.0%)	0.1379
Absent	90 (98.9%)	569 (100.0%)	

Table S13. LO HL Treated with Bevacizumab v LO NHW Treated with Bevacizumab.

WNT Pathway			
Gene	Late-Onset Hispanic/Latino Treated with Bevacizumab n (%)	Late-Onset NHW Treated with Bevacizumab n (%)	p-value
**AMER1 Mutation**			
Present	5 (11.6%)	22 (5.9%)	0.2686
Absent	38 (88.4%)	349 (94.1%)	
**APC Mutation**			
Present	34 (79.1%)	297 (80.1%)	1
Absent	9 (20.9%)	74 (19.9%)	
**AXIN1 Mutation**			
Present	0 (0.0%)	3 (0.8%)	1
Absent	43 (100.0%)	368 (99.2%)	
**AXIN2 Mutation**			
Present	0 (0.0%)	14 (3.8%)	0.3788
Absent	43 (100.0%)	357 (96.2%)	
**CTNNB1 Mutation**			
Present	3 (7.0%)	22 (5.9%)	0.735
Absent	40 (93.0%)	349 (94.1%)	
**GSK3B Mutation**			
Present	0 (0.0%)	1 (0.3%)	1
Absent	43 (100.0%)	370 (99.7%)	
**RNF43 Mutation**			
Present	1 (2.3%)	18 (4.9%)	0.7072
Absent	42 (97.7%)	353 (95.1%)	
**TCF7L2 Mutation**			
Present	5 (11.6%)	44 (11.9%)	1
Absent	38 (88.4%)	327 (88.1%)	
**TLE1 Mutation**			
Present	0 (0.0%)	0 (0.0%)	1
Absent	43 (100.0%)	371 (100.0%)	
**TLE2 Mutation**			
Present	0 (0.0%)	0 (0.0%)	1
Absent	43 (100.0%)	371 (100.0%)	

Table S14. LO HL Not Treated with Bevacizumab v LO NHW Not Treated with Bevacizumab.

WNT Pathway			
**Gene**	**Late-Onset Hispanic/Latino Not Treated with Bevacizumab n (%)**	**Late-Onset NHW Not Treated with Bevacizumab n (%)**	**p-value**
**AMER1 Mutation**			
Present	7 (6.3%)	146 (11.0%)	0.1693
Absent	104 (93.7%)	1184 (89.0%)	
**APC Mutation**			
Present	73 (65.8%)	999 (75.1%)	**0.03993**
Absent	38 (34.2%)	331 (24.9%)	
**AXIN1 Mutation**			
Present	3 (2.7%)	63 (4.7%)	0.4766
Absent	108 (97.3%)	1267 (95.3%)	
**AXIN2 Mutation**			
Present	8 (7.2%)	110 (8.3%)	0.8318
Absent	103 (92.8%)	1220 (91.7%)	
**CTNNB1 Mutation**			
Present	7 (6.3%)	93 (7.0%)	0.9371
Absent	104 (93.7%)	1237 (93.0%)	
**GSK3B Mutation**			
Present	1 (0.9%)	17 (1.3%)	1
Absent	110 (99.1%)	1313 (98.7%)	
**RNF43 Mutation**			
Present	20 (18.0%)	183 (13.8%)	0.2726
Absent	91 (82.0%)	1147 (86.2%)	
**TCF7L2 Mutation**			
Present	15 (13.5%)	225 (16.9%)	0.4283
Absent	96 (86.5%)	1105 (83.1%)	
**TLE1 Mutation**			
Present	0 (0.0%)	0 (0.0%)	1
Absent	111 (100.0%)	1330 (100.0%)	
**TLE2 Mutation**			
Present	0 (0.0%)	0 (0.0%)	1
Absent	111 (100.0%)	1330 (100.0%)	

Table S15. Mutational landscape of H/L and NHW early- and late-onset CRC with and without Bevacizumab treatment.

	Hispanic/Latino Samples				Non-Hispanic White Samples			
	Early-Onset		Late-Onset		Early-Onset		Late-Onset	
	Treated with Bevacizumab	Not Treated with Bevacizumab	Treated with Bevacizumab	Not Treated with Bevacizumab	Treated with Bevacizumab	Not Treated with Bevacizumab	reated with Bevacizuma	Not Treated with Bevacizumab
**AMER1**								
Frame Shift Deletion	0.0%	0.0%	40.0%	50.0%	12.5%	17.0%	9.1%	22.9%
Frame Shift Insertion	0.0%	10.0%	0.0%	0.0%	12.5%	3.8%	9.1%	7.2%
Missense Mutation	0.0%	20.0%	0.0%	25.0%	25.0%	37.7%	22.7%	36.7%
Nonsense Mutation	100.0%	70.0%	60.0%	25.0%	50.0%	41.5%	59.1%	33.1%
**APC**								
Frame Shift Deletion	35.9%	23.3%	30.2%	22.5%	32.2%	29.3%	29.1%	27.2%
Frame Shift Insertion	5.1%	6.8%	11.3%	5.4%	11.2%	8.5%	11.1%	10.2%
In Frame Deletion	0.0%	0.7%	0.0%	0.0%	0.0%	0.0%	0.0%	0.1%
In Frame Insertion	0.0%	0.0%	0.0%	0.0%	0.0%	0.0%	0.0%	0.1%
Missense Mutation	5.1%	15.1%	0.0%	4.5%	2.0%	8.5%	2.5%	6.1%
Nonsense Mutation	53.8%	52.7%	50.9%	63.1%	52.2%	51.4%	55.5%	54.3%
Splice Region	0.0%	0.0%	1.9%	0.0%	0.0%	0.0%	0.5%	0.0%
Splice Site	0.0%	1.4%	5.7%	4.5%	2.4%	2.2%	1.4%	1.9%
**AXIN1**								
Frame Shift Deletion	0.0%	0.0%	0.0%	50.0%	0.0%	0.0%	33.3%	26.0%
Frame Shift Insertion	0.0%	0.0%	0.0%	0.0%	0.0%	0.0%	0.0%	7.8%
Missense Mutation	0.0%	100.0%	0.0%	50.0%	0.0%	88.9%	66.7%	61.0%
Nonsense Mutation	0.0%	0.0%	0.0%	0.0%	0.0%	11.1%	0.0%	2.6%
Splice Site	0.0%	0.0%	0.0%	0.0%	0.0%	0.0%	0.0%	2.6%
**AXIN2**								
Frame Shift Deletion	0.0%	25.0%	0.0%	54.5%	0.0%	36.1%	7.1%	32.3%
Frame Shift Insertion	0.0%	25.0%	0.0%	9.1%	0.0%	16.7%	21.4%	20.0%
In Frame Deletion	0.0%	0.0%	0.0%	0.0%	0.0%	2.8%	14.3%	0.8%
In Frame Insertion	0.0%	0.0%	0.0%	0.0%	0.0%	0.0%	0.0%	4.6%
Missense Mutation	0.0%	25.0%	0.0%	27.3%	100.0%	36.1%	42.9%	37.7%
Nonsense Mutation	100.0%	0.0%	0.0%	9.1%	0.0%	2.8%	7.1%	0.8%
Splice Site	0.0%	25.0%	0.0%	0.0%	0.0%	5.6%	7.1%	3.1%
Translation Start Site	0.0%	0.0%	0.0%	0.0%	0.0%	0.0%	0.0%	0.8%
**CTNNB1**								
In Frame Deletion	0.0%	0.0%	0.0%	0.0%	10.0%	2.6%	9.1%	10.6%
Missense Mutation	50.0%	90.0%	66.7%	42.9%	30.0%	65.8%	50.0%	73.1%
Nonsense Mutation	0.0%	0.0%	0.0%	14.3%	0.0%	5.3%	0.0%	1.9%
Nonstop Mutation	0.0%	0.0%	0.0%	0.0%	0.0%	0.0%	0.0%	1.0%
Splice Site	50.0%	10.0%	33.3%	42.9%	60.0%	26.3%	40.9%	13.5%
**GSK3B**								
Frame Shift Insertion	0.0%	0.0%	0.0%	0.0%	100.0%	0.0%	0.0%	0.0%
Missense Mutation	0.0%	100.0%	0.0%	100.0%	0.0%	71.4%	100.0%	85.0%
Nonsense Mutation	0.0%	0.0%	0.0%	0.0%	0.0%	28.6%	0.0%	10.0%
Splice Site	0.0%	0.0%	0.0%	0.0%	0.0%	0.0%	0.0%	5.0%
**RNF43**								
Frame Shift Deletion	0.0%	50.0%	0.0%	50.0%	0.0%	55.7%	36.4%	60.0%
Frame Shift Insertion	0.0%	0.0%	0.0%	11.1%	66.7%	8.2%	22.7%	10.6%
Missense Mutation	0.0%	0.0%	0.0%	0.0%	0.0%	0.0%	0.0%	0.4%
Nonsense Mutation	0.0%	0.0%	0.0%	0.0%	0.0%	1.6%	0.0%	0.0%
Splice Site	0.0%	35.0%	100.0%	27.8%	0.0%	24.6%	18.2%	17.1%
Translation Start Site	0.0%	15.0%	0.0%	11.1%	33.3%	3.3%	22.7%	9.0%
**TCF7L2**								
Frame Shift Deletion	66.7%	47.4%	0.0%	37.5%	15.0%	30.5%	6.1%	43.7%
Frame Shift Insertion	0.0%	5.3%	20.0%	6.3%	25.0%	9.2%	8.2%	7.1%
In Frame Deletion	33.3%	0.0%	0.0%	0.0%	0.0%	0.8%	2.0%	2.0%
Missense Mutation	0.0%	31.6%	60.0%	50.0%	35.0%	34.4%	44.9%	32.3%
Nonsense Mutation	0.0%	5.3%	20.0%	0.0%	15.0%	15.3%	30.6%	9.1%
Splice Site	0.0%	10.5%	0.0%	6.3%	5.0%	9.9%	8.2%	5.9%
Translation Start Site	0.0%	0.0%	0.0%	0.0%	5.0%	0.0%	0.0%	0.0%
**TLE1**								
Missense Mutation	0.0%	100.0%	0.0%	0.0%	0.0%	0.0%	0.0%	0.0%
**TLE2**								
Frame Shift Deletion	0.0%	100.0%	0.0%	0.0%	0.0%	0.0%	0.0%	0.0%

(a)

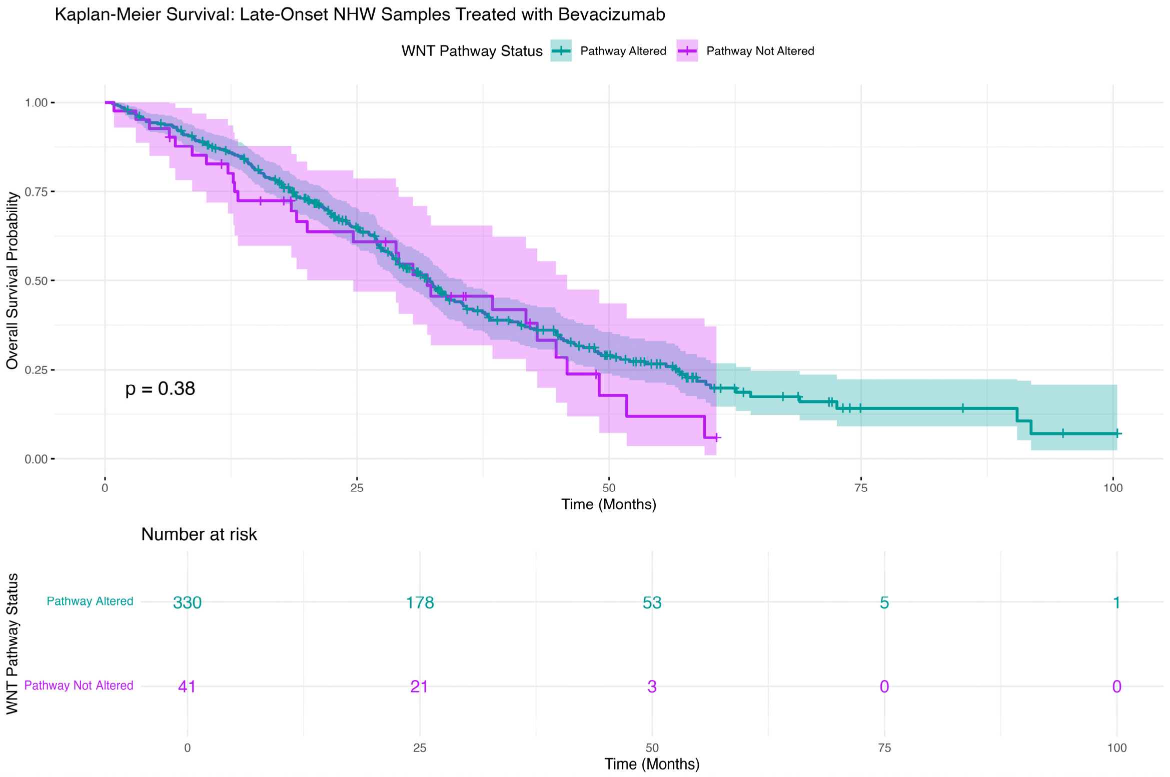

(b)

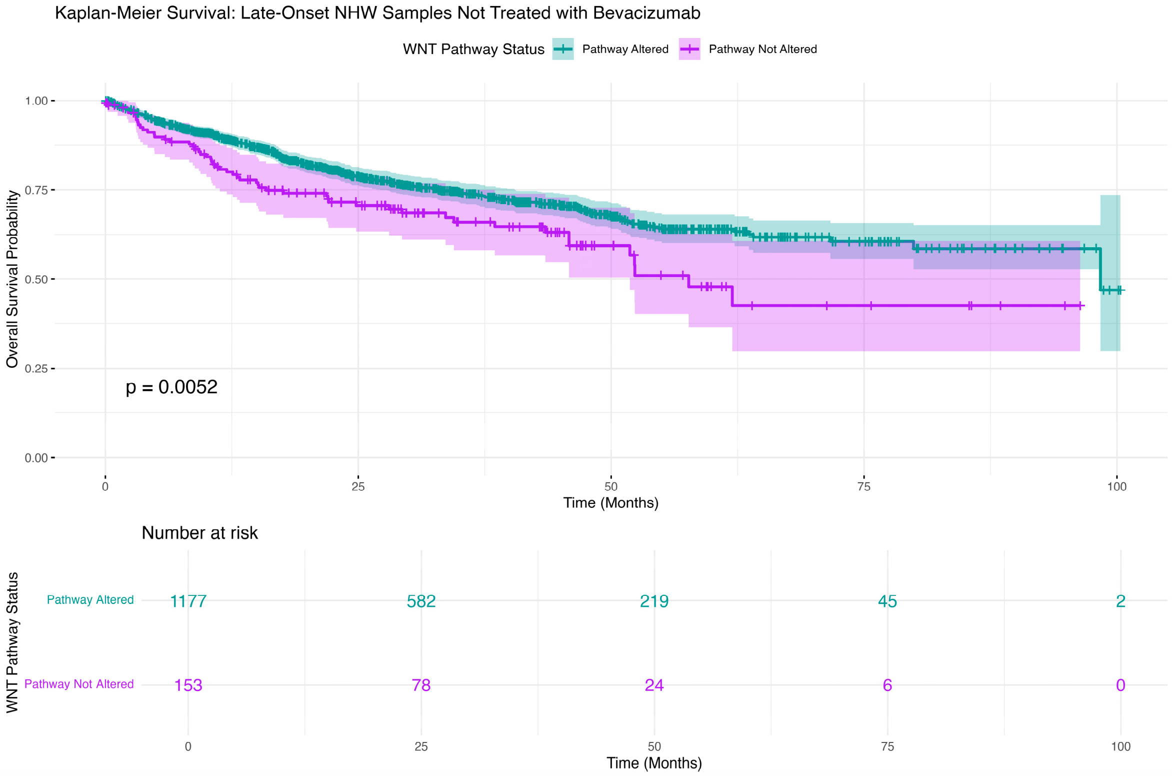

Figure S2. Kaplan-Meier survival analysis according with WNT pathway alteration status across age and Bevacizumab treatment status in colorectal cancer. Overall survival analyses were performed across patient groups: (a) Late-Onset NHW patients treated with Bevacizumab, and (b) Late-Onset NHW patients not receiving Bevacizumab.

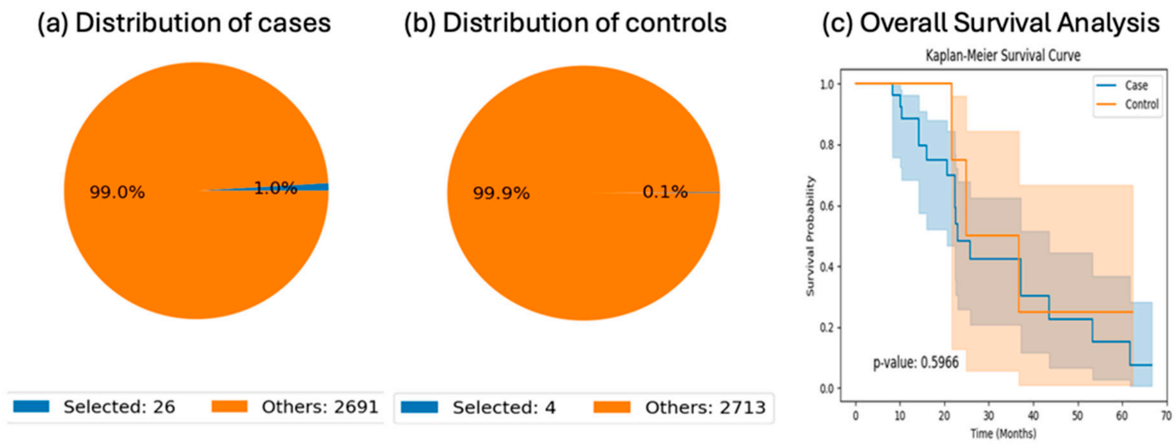

Figure S3. AI-assisted survival analysis of WNT pathway status in Bevacizumab-treated early-onset Hispanic/Latino colorectal cancer. This figure summarizes the construction of a clinically and molecularly defined cohort and the corresponding overall survival analysis in early-onset Hispanic/Latino colorectal cancer patients exposed to Bevacizumab. Using AI-HOPE-WNT–supported cohort stratification, patients were grouped according to WNT pathway alteration status, yielding 26 WNT-altered cases and 4 WNT–unaltered controls. Panels (a) and (b) illustrate the proportion of selected samples relative to the full dataset, underscoring the limited size and rarity of this treatment- and ancestry-specific subgroup. Panel (c) shows Kaplan–Meier estimates of overall survival comparing WNT-altered and non-altered tumors. Survival trajectories were largely overlapping, with no statistically significant difference observed between groups (log-rank p = 0.5966). Shaded regions represent 95% confidence intervals. Collectively, this analysis highlights both the feasibility of AI-enabled, multi-parameter cohort definition in underrepresented populations and the absence of a detectable survival association between WNT pathway status and Bevacizumab-treated EOCRC in this limited sample.

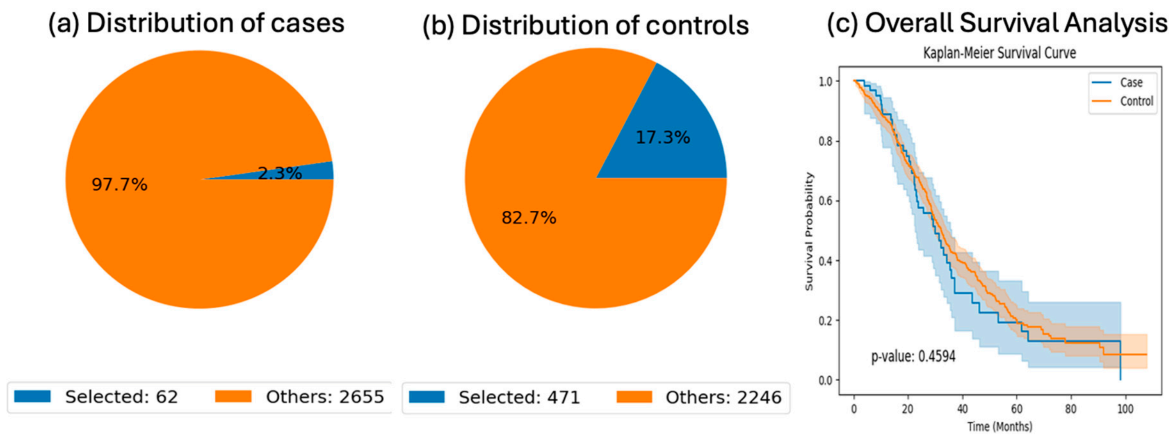

Figure S4. Ethnicity-stratified overall survival analysis among Bevacizumab-treated colorectal cancer patients with WNT pathway alterations. This figure depicts an AI-assisted comparison of overall survival between Hispanic/Latino (H/L) and non-Hispanic White (NHW) colorectal cancer patients receiving Bevacizumab who harbor WNT pathway alterations. AI-HOPE-WNT was used to harmonize clinical, treatment, and genomic data and to define ancestry-specific cohorts, identifying 62 H/L cases and 471 NHW controls. Panels (a) and (b) illustrate the relative representation of each ancestry group within the full analytic dataset, highlighting the limited size of the WNT-altered, Bevacizumab-treated H/L subgroup compared with NHW patients. Panel (c) presents Kaplan–Meier estimates of overall survival by ethnicity, demonstrating largely overlapping survival curves with no statistically significant difference between groups (log-rank p = 0.4594). Shaded areas indicate 95% confidence intervals. Together, these results indicate no detectable ethnicity-associated survival difference in this treatment- and pathway-defined population and illustrate the utility of AI-enabled clinical informatics tools for scalable, ancestry-aware survival analyses in precision oncology.

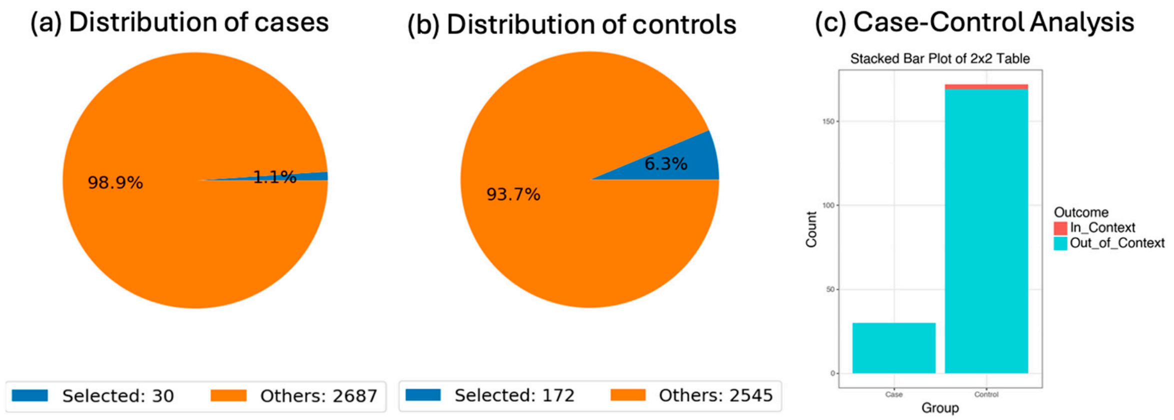

Figure S5. **A**ncestry-based comparison of RNF43 mutation prevalence in Bevacizumab-treated early-onset colorectal cancer. This figure presents a case–control analysis examining differences in RNF43 mutation frequency between early-onset Hispanic/Latino (H/L) and non-Hispanic White (NHW) colorectal cancer patients, all of whom received Bevacizumab. AI-HOPE-WNT–assisted cohort definition identified 30 H/L cases and 172 NHW controls meeting age, ancestry, and treatment criteria. Panels (a) and (b) display the proportion of RNF43-mutated ("in-context") tumors relative to the full dataset in each group, illustrating the low overall prevalence of RNF43 alterations in both populations (1.1% in H/L and 6.3% in NHW). Panel (c) summarizes mutation status using a 2×2 contingency framework. Fisher's exact testing yielded no statistically significant difference in RNF43 mutation prevalence between ancestries (odds ratio = 1.0; 95% CI, wide; $p > 0.05$). These results indicate comparable RNF43 alteration rates across H/L and NHW patients within this treatment-defined early-onset cohort and demonstrate the utility of AI-enabled clinical informatics approaches for rapid, ancestry-aware genomic comparisons in precision oncology.

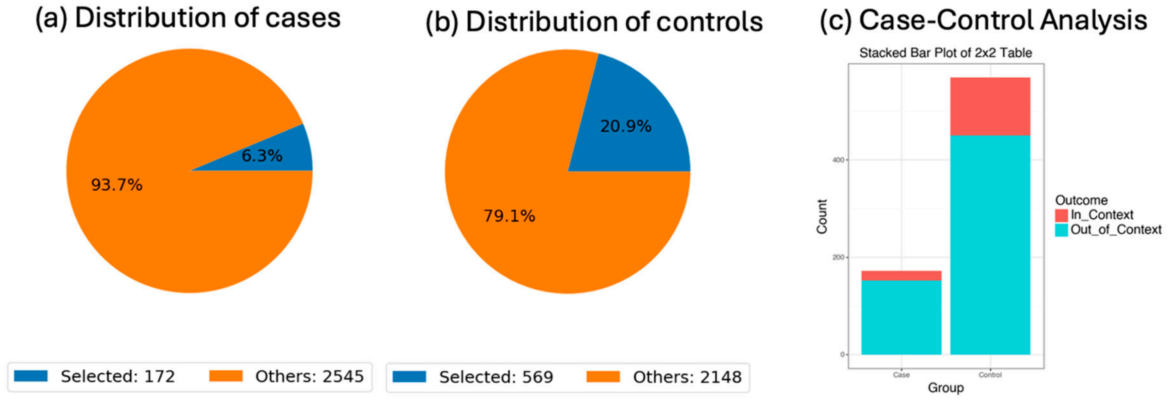

Figure S6. Bevacizumab-associated differences in TCF7L2 mutation frequency among early-onset non-Hispanic White colorectal cancer patients. This figure depicts a treatment-stratified genomic comparison assessing whether exposure to Bevacizumab is associated with variation in TCF7L2 mutation prevalence in early-onset non-Hispanic White (NHW) colorectal cancer. AI-HOPE-WNT–assisted cohort construction identified 172 Bevacizumab-treated cases and 569 untreated controls meeting age and ancestry criteria. Panels (a) and (b) illustrate the relative proportion of TCF7L2-mutated tumors within each cohort, showing a lower mutation frequency in treated patients (6.3%) compared with untreated patients (20.9%). Panel (c) summarizes mutation status using a 2×2 contingency framework, visualized as a stacked bar plot. Statistical evaluation with Fisher's exact test demonstrated a significant difference between groups ($p = 0.009$). The estimated odds ratio (0.498; 95% CI, 0.299–0.827) indicates reduced odds of TCF7L2 mutation among Bevacizumab-treated early-onset NHW patients. Together, these results suggest a treatment-associated shift in the mutational landscape of WNT pathway regulators and illustrate the utility of AI-enabled clinical informatics approaches for identifying therapy-linked molecular patterns in colorectal cancer.

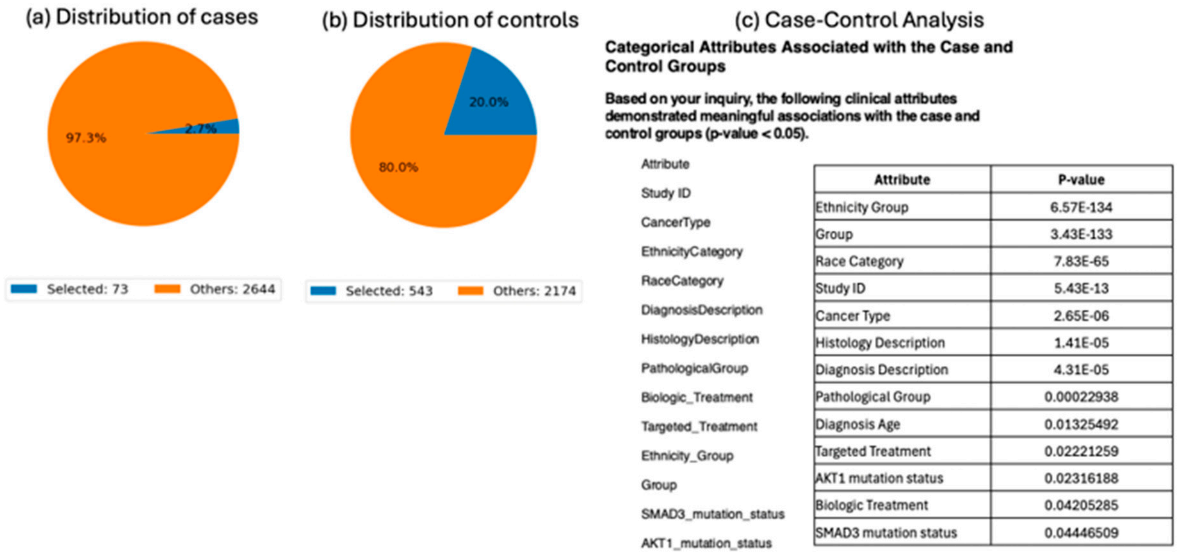

Figure S7. Ethnicity-associated clinical and molecular features among Bevacizumab-treated colorectal cancer patients. This figure summarizes an AI-enabled comparison of clinical and molecular attributes between Hispanic/Latino (H/L) and non-Hispanic White (NHW) colorectal cancer patients who received Bevacizumab. Using AI-HOPE-WNT–assisted cohort definition, 73 H/L cases and 543 NHW controls were identified based on ethnicity and treatment exposure. The pie charts illustrate the relative representation of each group within the overall dataset, highlighting the smaller proportion of Bevacizumab-treated H/L patients. Automated categorical association testing was then applied to screen a broad set of clinical, diagnostic, and genomic variables. Several attributes demonstrated statistically significant differences between groups ($p < 0.05$), including demographic classifications, tumor diagnostic and histologic descriptors, age at diagnosis, treatment-related variables, and selected pathway-linked gene alterations (e.g., AKT1 and SMAD3). Together, these results demonstrate the capacity of AI-enabled clinical informatics workflows to rapidly identify ancestry-associated clinical and molecular patterns within treatment-defined populations, supporting hypothesis generation for disparity-aware precision oncology.
